# Whole genome-scale assessment of gene fitness of *Novosphingobium aromaticavorans* during spaceflight

**DOI:** 10.1186/s12864-023-09799-z

**Published:** 2023-12-16

**Authors:** Gayatri Sharma, Peter C. Zee, Luis Zea, Patrick D. Curtis

**Affiliations:** 1 Department of Biology, University of Mississippi, 402 Shoemaker Hall, University, MS 38677 USA; 2 Aerospace Engineering Sciences, University of Colorado Boulder, Boulder, CO 80303 USA

**Keywords:** Microgravity, *Novosphingobium*, Genome fitness, Comparative TnSeq, Fluid processing apparatus, International space station

## Abstract

In microgravity, bacteria undergo intriguing physiological adaptations. There have been few attempts to assess global bacterial physiological responses to microgravity, with most studies only focusing on a handful of individual systems. This study assessed the fitness of each gene in the genome of the aromatic compound-degrading Alphaproteobacterium *Novosphingobium aromaticavorans* during growth in spaceflight. This was accomplished using Comparative TnSeq, which involves culturing the same saturating transposon mutagenized library under two different conditions. To assess gene fitness, a novel comparative TnSeq analytical tool was developed, named TnDivA, that is particularly useful in leveraging biological replicates. In this approach, transposon diversity is represented numerically using a modified Shannon diversity index, which was then converted into effective transposon density. This transformation accounts for variability in read distribution between samples, such as cases where reads were dominated by only a few transposon inserts. Effective density values were analyzed using multiple statistical methods, including log_2_-fold change, least-squares regression analysis, and Welch’s t-test. The results obtained across applied statistical methods show a difference in the number of significant genes identified. However, the functional categories of genes important to growth in microgravity showed similar patterns. Lipid metabolism and transport, energy production, transcription, translation, and secondary metabolite biosynthesis and transport were shown to have high fitness during spaceflight. This suggests that core metabolic processes, including lipid and secondary metabolism, play an important role adapting to stress and promoting growth in microgravity.

**Supplementary Information:**

The online version contains supplementary material available at 10.1186/s12864-023-09799-z.

## Introduction

Since the 1960s, it has been observed that bacteria have the capability to survive spaceflight [1]. In the closed system of space-faring vehicles, bacteria experience constant weightlessness, a reduction in fluid perturbation leading to a dependence on molecular diffusion, and chemical alterations in the cellular microenvironment, all of which create a distinct environment from what is observed on Earth [2–4]. Bacteria have been shown to physiologically respond to spaceflight conditions, illuminating the impact of microgravity on living systems and also highlighting the adaptability of microbes to extreme conditions on Earth and beyond. Such responses include evolved increased resistance to antibiotics [5–8], increased virulence [6, 9–12], enhanced growth [13–16], changes in cell size [17], altered production and yield of secondary metabolites [18–21], changes in biofilm formation [22–25], altered motility and chemotaxis [26–29], and uniquely impacted stress responses [30]. However, such responses vary among different bacteria. Enhanced growth rates were observed for *Escherichia coli, Bacillus subtilis, Salmonella typhimurium*, and *Pseudomonas aeruginosa* in the spaceflight environment [13–16, 22, 31, 32], while other studies on *E. coli, B. subtilis*, and *Staphylococcus aureus* [27, 28, 33–36] found no change in growth rate. Similarly, increased stress tolerance has been observed in *P. aeruginosa* [24, 26] and *E. coli* [8], along with upregulation of genes associated with oxidative and osmotic stress in *Rhodospirillum rubrum* [37], while *S. aureus* showed decreased stress response [38]. These diverse responses of bacteria highlight the need to study more microbial species to understand how microgravity and low-shear stress can affect different bacteria and advance our understanding of their responses to spaceflight.

Most studies conducted in space have primarily focused on either opportunistic pathogens [10, 39–42] or model organisms [43, 44]. However, the microbiome of the International Space Station (ISS) is diverse in composition and exhibits variation in diversity over time and locations aboard the station [45, 46]. Around 12,554 distinct microbial species have been found aboard ISS, with the most abundant human-associated Actinomycetales (18.3%) and Bacillales (14%) to less abundant Sphingomonadales (0.9%) and Sphingobacteriales (0.8%) [47]. Since each bacterial species can exhibit their own unique response to microgravity [48], and bacterial adaptation responses can impact spaceflight functions leading to both detrimental effects and beneficial outcomes [45, 49, 50], it is crucial to study diverse bacteria. This study focuses on *Novosphinogobium aromaticivorans*, a Gram negative Alphaproteobacterium, that can degrade aromatic compounds with potential benefits in the de-polymerization of lignin [51], which can be leveraged in turnover of recalcitrant biological material for composting or biofuel production. Such activities can be useful as humans push to have longer term colonization in altered gravity environments which will require local plant cultivation and biomass turnover. This organism has already been detected in the clean room facilities utilized for packaging cargo sent to the ISS [52], so it has the potential to be a shipboard contaminant of spaceflight expeditions. A genome-level gene fitness study will help mechanistically understand how *N. aromaticivorans* responds to microgravity and investigate how much such responses are unique compared to examined model or pathogenic bacteria.

While many previous studies have focused on investigating specific physiological systems or functions, when considered together they indicate that physiological adaptation and survival under microgravity involves global-scale changes in microbial physiology that are very poorly understood at the genetic level. A few studies in the past have attempted to investigate global responses of bacteria to microgravity by examining the changes in gene expression primarily through microarrays and real-time quantitative PCR [10, 39, 40]. Some studies investigated phenotypic variation after spaceflight followed by transcriptomics and proteomics which provided insights into significant changes in their metabolic profiles [37, 41]. Transcriptomics, although comprehensive in capturing changes in transcript levels is limited to specific time frames. It may miss important transcriptional changes occurring outside the experimental duration. Similarly, proteomics faces challenges in fractionating bacteria for analysis, particularly with Gram-negative strains [26, 53, 54]. To gain a deeper understanding of the genetic mechanisms underlying these global physiological changes, a more comprehensive investigation of the bacterial genome is necessary. This study is the first of its kind, aiming to comprehensively understand global-scale genome fitness under real microgravity using Comparative TnSeq. This approach combines saturating transposon mutagenesis with growth under different conditions, followed by high throughput sequencing to identify genes with unique representation/diversity of transposon inserts per gene per condition over generations. Thus, Comparative TnSeq reveals the fitness contribution of a given gene in a given condition. Additionally, this study developed a novel TnSeq analysis methodology, borrowing tools commonly used to measure biodiversity in ecological studies. This study also applies microgravity to cultures by growing them aboard the International Space Station, which provides an ideal platform for studying the impact of real microgravity on bacterial responses as no ground-based facility can fully simulate actual spaceflight [18, 52, 55] The results of this study can inform the design of mechanisms for intelligently manipulating deleterious impacts that bacteria can have on the systems aboard the ISS.

## Experimental procedures

### Transposon library preparation

The EZ-Tn5 transposon mutagenized library of *Novosphingobium aromaticivorans* DSM 12,444 used in this study was prepared following the protocol of [56], which is based on the protocol of [57]. *N. aromaticivorans* was cultured in 2 X PYE media (4 g/L bactopeptone, 2 g/L yeast extract, 0.014 g/L CaCl_2_ * 2 H_2_O, 0.6 g/L MgSO_4_ * 7 H_2_O,) [58] to an OD_600_ of approximately 1.0, and the cells were harvested by centrifugation (12,000 x g, 5 min, 4 °C). The cells were then resuspended in 25 ml ice-cold water and centrifuged, and this process was repeated. Finally, the cells were resuspended in 250 µl ice-cold water. EZ-Tn5 transposome (1 µl, Epicentre) was added to the cells and mixed by gentle pipetting. The cell and transposome mixture was aliquoted into five chilled electroporation cuvettes (0.1 cm) and subjected to electroporation (1.5 kV, 25 µF, 400Ω). Each electroporation was resuspended in 1.0 ml 2X PYE and incubated with shaking at 30 °C for 3 h. The cultures were plated 3 × 350 µl onto 150 mm diameter PYE (2 g/L peptone, 1 g/L yeast extract, 0.3 g/L MgSO4 * 7 H_2_O, 15 g/L agar) [58] agar plates supplemented with kanamycin (20 µg/ml). The plates were incubated at 30 °C for 2 days, and the resulting colonies were harvested as a pool. Multiple aliquots from each pool were frozen at -80 °C in 10% DMSO for storage.

### Assessment of viability and contamination in transposon library

The frozen *N. aromaticivorans* Tn5 mutant library was assessed for viability loss resulting from storage and repeated thawing and refreezing, and to check for contamination. Serial dilutions of the mutant library were prepared using 2X PYE liquid media. A 10 µl volume from the library was used, and four 100-fold serial dilutions were performed. A 100 µl aliquot from each dilution was spread plated onto PYE agar plates supplemented with kanamycin (20 µg/ml) to select for mutants with transposon insertions. Three replicates of each dilution were used. The plates were incubated at 30 °C for 2 days to allow colony appearance. The number of colonies were counted manually and the CFU/ml was calculated. The plates were examined for contamination, and the extent of contamination was determined based on the number of contaminant colonies on each dilution plate.

### Optimization of the pre-culturing stowage and culture conditions

**Fig. 1:**
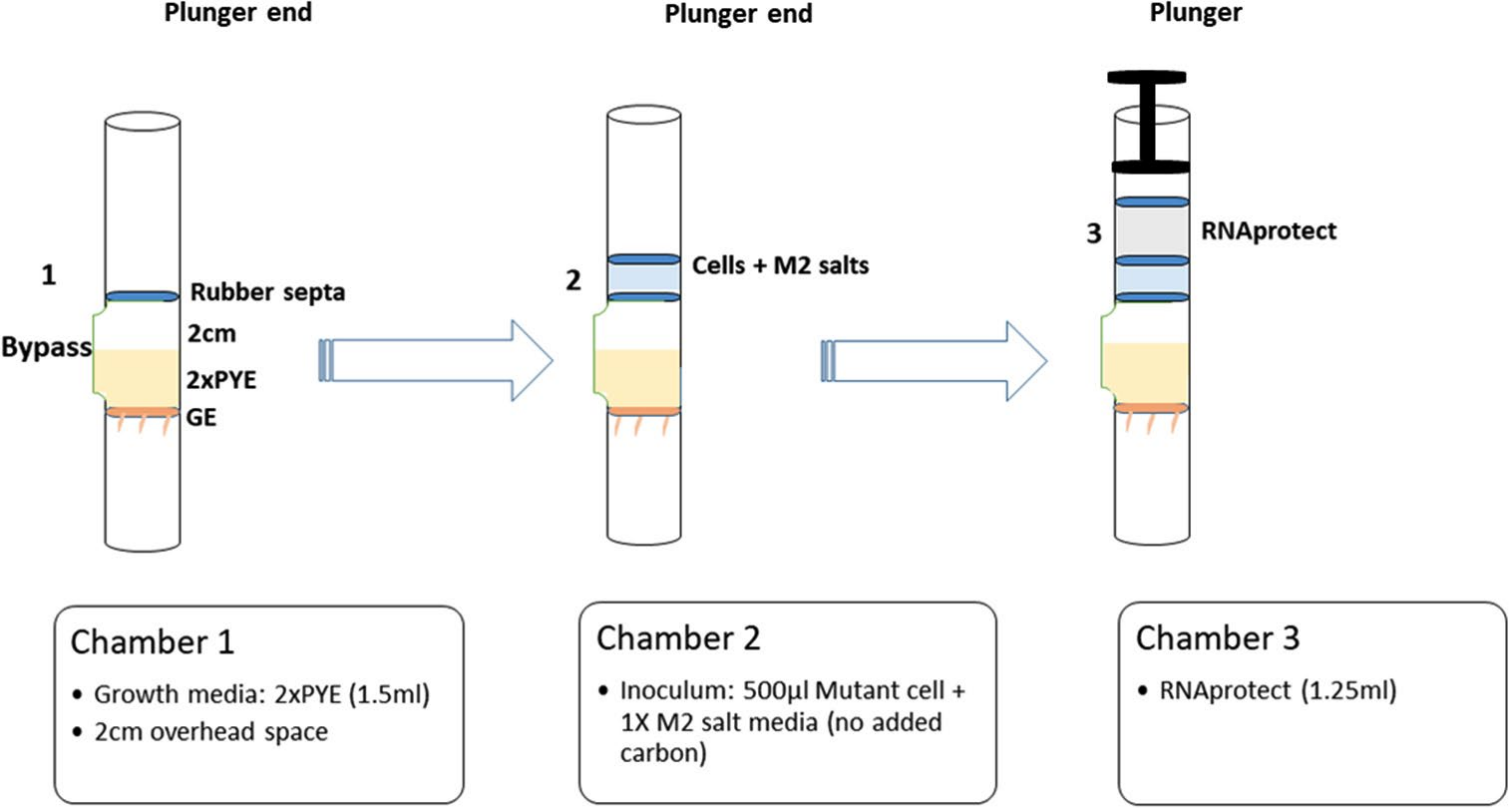
Schematic of FPA assembly used to culture *N. aromaticivorans* in spaceflight. Chamber 1 contained 1.5 ml of 2XPYE with an additional 2 cm of airspace and was capped on the non-plunger end by a gas exchange membrane. Chamber 2 contained 1 ml of *N. aromaticivorans* TnSeq library inoculum in M2 salts. Chamber 3 contained 1.25 ml of RNAprotect fixative. Chambers were mixed by pressing the terminal septum with a plunger. When an internal septum reached the bypass, the liquid from the upper chamber was transferred to the lower chamber and mixed by shaking by hand

Culturing of libraries under experimental conditions was performed using the Fluid Processing Apparatus (FPA, Bioserve Space Technologies). This apparatus consists of a cylindrical tube with a bypass channel in one side. Chambers were created by addition of moveable silicone rubber septa, and the terminal growth chamber was capped with a gas exchange membrane as illustrated in Fig. 1. Pressing a plunger at one end moves the septa until one intersects the bypass chamber, at which point the liquid from the upper chamber passes through the bypass channel into the lower chamber, thus mixing the samples. Glass barrels and rubber stoppers were coated with a silicone lubricant (Sigmacote; Sigma), off-gassed, and autoclaved separately before assembly. FPAs were stored in the Group Activation Pack (GAP), which allowed simultaneous inoculation of several FPAs in each GAP by use of a hand crank. Both FPAs and GAPs have been utilized in multiple previous spaceflight studies [10, 15, 22, 39]. For flight experiments, a 3-chamber liquid holding approach in the FPA was used (Fig. 1). The first chamber (growth chamber) contained 1.5 ml 2X PYE + Kanamycin (20 µg/ml) with 2 cm air space. The air space provided additional oxygen for growth, as previous setups without this air space resulted in dramatically low biomass, presumably due to oxygen restriction. The second chamber (inoculum) contained 500 µl 1X M2 salts (1.74 g/L Na_2_HPO_4_, 1.06 g/L KH_2_PO_4_, 0. 5 g/L NH_4_Cl, supplemented with 123 g/L MgSO_4_ (500 mM), 7.35 g/L CaCl2 (50 mM) and 0.278 g/L FeSO_4_/EDTA (1 mM) [58] and 500 µl *N. aromaticivorans* TnSeq library cells diluted in 1X M2 salts to 5 × 10^5^ viable cells/ml. The third chamber (preservative) contained 1.25 ml RNAprotect (Qiagen). Eight FPAs were assembled, four used for launch and four used for on-ground control.

Transportation to the International Space Station and other pre-culturing delays required extended cold storage times of pre-culturing samples. To assess the impact of pre-culturing stowage on the viability of the bacterial samples, a temperature tolerance test was conducted. Cells were suspended in carbon-deficient 1X M2 salts and stored at temperatures of 2 °C, 4 °C, and 6 °C for 21 days in FPAs. Three replicates were prepared for each temperature. To monitor cell count and viability, one culture replicate from each temperature was removed every 7 days over the 21-day period. The cultures were serially diluted and plated on PYE agar plates, which were then incubated at 30 °C and CFU/ml was calculated.

A previous study indicated that a growth of 5 generations in a minimum volume of 1.5 ml was adequate to observe differences in gene fitness under different conditions using TnSeq [59]. Furthermore, it has been shown that a minimum of 5 generations is required for the elimination of mutations with lower fitness, while 15 generations of growth still avoids the loss of mutant diversity due to random chance [60, 61]. Based on this information, the culture volume and incubation time were determined to provide optimal growth generations and yield sufficient biomass for DNA sequencing. Preliminary growth data was generated by culturing library cells inside FPAs on Ground, and growth was monitored by measuring turbidity and cell count. At different time intervals, samples were extracted from the cultures and CFU/ml was determined.

### Transportation and culture activation

FPAs were prepared and assembled at the Eastern Virginia Medical School (EVMS). Hardware was provided by Bioserve Space Technologies and autoclaved in the EVMS facility. Assembly was performed under the supervision of Bioserve Space Technologies. Following the assembly process, all FPAs were handed over to Bioserve Space Technologies 24 h before the launch for pressure check and assembly into GAPs.

The initial launch attempt scheduled for September 27, 2020 was scrubbed due to technical reasons and rescheduled for October 2, 2020. During this time, the samples were stored at 4 °C. The second launch attempt was successful and the samples were successfully launched into space onboard the Antares launch vehicle. The Ground samples were sent back to the laboratory at the University of Mississippi and stored at 4 °C until culture activation. The rocket berthed with the International Space Station on October 5, 2020. On October 7, 2020, the samples were activated by mixing the inoculum with the growth media. Astronaut Chris Cassidy performed the mixing by cranking the GAPs, pushing the rubber septa of 8 enclosed FPAs, which allowed the cells in Chamber 2 to mix with the growth media in Chamber 1. Ground control samples were inoculated at the same time in the same manner. Thus, cells were stored at 4 °C for a total of 11 days until the 30 °C incubation was started on both ISS and Ground. A coordinated operations timeline was recorded for both in-flight and on-ground incubation (Supplementary Data_S1). Ground controls were incubated at 30 °C without shaking with GAPs placed in a horizontal orientation. This was done to prevent nutrient limitation that could occur due to sedimentation in Chamber 1. Similarly, onboard the space station GAPs were incubated in the SABL S/N 2 incubator pre-conditioned to 30 °C. Following the incubation duration of 5 days, fully turbid cultures, indicating proper growth, were terminated under both conditions simultaneously. Cultures were terminated by mixing with RNAprotect fixative (Chamber 3) and stored at -80 °C. The GAPs returned on SpaceX CRS-21 Dragon, which splashed down on January 12, 2021. The frozen GAPs were at the Kennedy Space Center a few hours later and placed in a -80 °C freezer. They were received at the University of Mississippi on January 15, 2020, and stored in a -80 °C freezer until DNA extraction was performed.

### Genomic DNA extraction and transposon sequencing

Cultures were thawed and genomic DNA was extracted using the Qiagen Maxi Prep Kit following the manufacturer’s protocol. The harvested DNA was further purified using the Zymo ZR-96 DNA Cleanup and Concentrator™-5 kit following the manufacturer’s protocol. A minimum of 100 ng of DNA in 6 µl, resulting in a minimum concentration of 20 ng/µl in nuclease-free water, was used for sequencing, while the remaining DNA was stored at -80 °C.

The library preparation and sequencing was based on general protocol followed in [62]. Library preparation, sequencing, and sequence analysis were performed by the Indiana University Center for Genomics and Bioinformatics. Nextera libraries were prepared for each sample following the Illumina Nextera DNA Flex Library Prep protocol, now referred to as Illumina DNA Prep. Libraries were quantified using an Agilent 4200 TapeStation and diluted to 1 nM as a template for transposable element (TE) library construction via Nested PCR. Multiplex PCR reactions were then performed using primers specific to the TE and the index i5 primer.

To amplify the fragments with the TE-specific genomic context, two separate multiplex PCR reactions were performed simultaneously using TE-specific primers in combination with the Illumina i5 primer. Two sets of primers (forward and reverse) were designed for the two separate PCR reactions. In these PCR reactions (10 cycles), amplification was produced using a combination of TE-specific primers and the i5 primer (Supplementary Data_S2). Amplification of the junctions between the TEs and the surrounding genomic DNA at both the 5’ end and the 3’ end of the TE was performed. Both reactions involved amplification with the i5 primer oriented in either direction with respect to the TE, in combination either with TE-specific Reverse and Forward primers, respectively. The i5 primer was used in combination with TE-specific reverse primers for amplifying the 5’ end junctions and TE-specific forward primers for amplifying the 3’ end junctions. The forward and reverse TE-specific primers and their respective annealing temperatures for these PCR reactions (PCR1) are listed in the Supplementary Data_S2. A second nested PCR (10 cycles) was performed to enrich for the TE-gDNA junctions, utilizing nested primers that bind within the TE region and the i5 adaptor. The products of PCR1 were used as templates for the second nested PCR (PCR2). Both TE-specific nested PCR primers contained a specific overhang region (5’-GTTCAGACGTGTGCTCTTCCGATCT-3’) to facilitate the addition of the index in the next PCR step. This PCR step amplified the DNA fragments containing the 5’ (amplified using the reverse primer) or 3’ (amplified using the forward primer) flanking regions of the TEs. The TE-Nest primers for PCR2 are listed in the Supplementary Data_S2.

The final step was the Index PCR, which added the i7 adaptor and index using the NEBNext® Multiplex Oligos for Illumina kit. The products of the Nest PCRs containing either the TE 5’ or 3’ flanking regions were combined and used as the template for the Index PCR. The Index PCR was performed using the Illumina i5 primer and the NEBNext® Multiplex Oligos to add the i7 adaptor and index. Phusion polymerase was used for all PCR reactions. The final libraries were analyzed using the Agilent 4200 TapeStation, purified with 0.8X AMPure XP beads, washed with 80% ethanol twice, and eluted with elution buffer. The libraries were loaded on NextSeq 500/550 High Output (75 cycle) v2.5 flow cells configured to generate 75 bp paired-end reads. The demultiplexing of the reads was performed using bcl2fastq version 2.20.0.

The sequencing data was then processed, including culling reads lacking the transposon sequence at the start and trimming transposon sequences using Cutadapt (v3.5) from relevant reads before mapping using these non-default parameters: --trimmed-only -g GGTTGAGATGTGTATAAGAGACAG. The mapping process involved aligning the sequences to reference genetic elements, specifically *Novosphingobium aromaticivorans* DSM 12444 (CP000248.1) and its plasmids pNL1 (CP000676.1) and pNL2 (CP000677.1). The mapping tool utilized was bowtie2 (v2.3.5.1) which used default parameters with added flags: --no-unal --no-mixed [61, 62]. Quality control and preprocessing of generated FASTQ files to generate clean data for downstream analysis was preprocessed using Fastp (v0.23.2). The insertion site for each read was determined based on the coordinate to which the 5’ end of the read mapped.

### Comparative TnSeq analysis

Gene fitness comparison between Ground and ISS conditions was performed by a novel approach. Transposon insertion sites and read counts were used to calculate the Shannon diversity index of each ORF. The Shannon diversity index, also called the Shannon–Weiner index (H′), is a common approach to quantify biodiversity in community ecology [63, 64] taking into account data on both the number of unique species (richness) and relative abundance (evenness). In this analysis, unique transposon insertion sites were substituted for species, and read counts per insertion site were substituted for number of individuals per species. Each replicate per condition was treated as an individual sample and each gene was considered a ‘community’ with unique transposon insertion sites. To mitigate potential artifacts that could inflate transposon representation and impact fitness evaluation, precautionary measures were taken by excluding 20% of the coding sequence from both the 5’ and 3’ ends of each gene. This trimming aimed to exclude insertions at these ends that reflect improperly-annotated start sites and that do not reflect true functional disruption respectively. Also, to account for PCR generated biasness, the read count data was normalized using DESeq2 package [65, 66]. The Shannon diversity index of each gene for all the replicates was calculated as:

H’ = –Σ_i=1_ (P_i_) log (P_i_).

where, H’ is the transposon diversity index per gene and *P*_*i*_ is the proportion of counts for each unique insert belonging to a gene *i* [67].

Comparing diversity indices may not accurately measure proportional changes of true diversity between conditions [68], because indices fail to satisfy the replication principle or the “doubling property” [67]. The doubling property suggests that doubling the number of equally common species should result in double the index value. However, this principle may not hold true with Shannon indices. Simply doubling the difference in Shannon diversity index between two conditions does not necessarily indicate a two-fold increase in species count. Consequently, relying solely on index values to observe the difference does not accurately represent the actual gain or loss between conditions. This issue results in challenges comparing H’ values among genes between conditions. To address this limitation and scaling issue, it is often recommended to transform the unit-less Shannon indices by calculating the exponent of the Shannon index (‘D). This transformation provides intuitive units of density measurement that are comparable [69], and enables a more meaningful interpretation of the relative representation of transposon inserts in a gene per condition.

‘D = exp (-∑(Pi * log(Pi))).

The generated ‘D is considered the effective number of sites, which represents the number of equally abundant or equally frequent transposon sites that would be needed to have the same level of indices as observed [69–72]. The applied approach accounted for variability in read distribution between samples, such as cases where some reads were dominated by only a few transposon inserts while in others read distribution were more evenly distributed. Effective number of transposon sites were further converted to an ‘effective density’ (ED) as follows

ED = ’D/(gene length).

Thus, effective density per gene was used as a measure to see the fitness difference between conditions, taking into account the distribution of transposon inserts within each gene. The lower the effective density within a gene under specific conditions, the lower the fitness of that gene is considered, thereby highlighting the gene’s significance in those conditions.

Multiple tests were employed to detect genes with significant differences in the effective densities across conditions (i.e., Ground vs. ISS). All the analyses were conducted in R version 4.2.1, and ‘vegan’ and ‘ggplot’ packages were used to calculate Shannon indices and to generate figures. Common statistically significant genes detected from all three tests were considered crucial for growth and survival at microgravity condition on ISS.

#### Log_2-_-fold change

Log_2-_fold change of average effective density per gene ratio was calculated as follows.

log_2_ (average effective density (G) / average effective density (I)).

where the average effective density was calculated by averaging the effective density for a given gene from all the replicates in a condition. The resulting ratio represents the fold change in a gene’s average effective density between the Ground (G) and ISS (I) replicates. The resulting ratio was then log_2_-transformed and plotted across the genome.

A fold change of 1 or -1 indicates a two-fold difference in effective density, which suggests a potentially significant difference between the conditions. On the other hand, a fold change of 0 for a gene suggests no potentially significant difference in effective density between the conditions. Those genes that had a log2-fold change of more than 1 were considered to have provided high fitness to growth in microgravity.

#### Linear regression

By regressing the average effective density values of the two conditions against each other, linear regression analysis was performed to identify influential genes based on a Cook’s distance outlier analysis. Cook’s distance was calculated for each gene as a measure to evaluate the impact of removing a specific gene from the dataset on the estimated regression coefficient. Genes that exhibit a significant change in the regression coefficient were considered influential. These influential points had the potential to affect the fit of the regression. Thus, Cook’s distance quantified the influence of a gene’s effective density on the estimated regression coefficients or the overall fit of the regression model.

A threshold value of Cook’s distance (> 4/n, where n is the number of genes in the data) was used to determine influential or outlier genes [73, 74]. A large Cook’s distance value for a gene indicated substantial impact on the estimated coefficients or a significant effect on the overall fit of the regression model, suggesting a strong influence of that gene’s effective density in the comparison between the conditions. These influential genes with their substantial effect on the regression analysis might hold potential significance for microgravity.

#### Welch’s t-test

The Welch’s t-test was utilized to compare the mean effective density of genes between conditions across the entire genome to identify genes with significant differences. Leveraging replicates helped minimize potential biases or random variations present in individual samples, and increased the statistical power and confidence in the analysis resulting in more reliable estimates of transposon diversity. Two cutoffs were applied for significance: p ≤ 0.01 (1% significance level) and p ≤ 0.05 (5% significance level). These cutoffs were used to determine genes that exhibited statistically significant differences in effective density between the conditions. The selection of these cutoffs aimed to strike a balance between controlling Type I errors (false positives) and maintaining reasonable statistical power respectively. Genes with p-values less than or equal to 0.01 were considered strongly significant.

Overall, this proposed novel comparative TnSeq analysis has been designed to accommodate both unique insertions and read counts while leveraging multiple replicates. Given the statistical methods used for analysis in this approach, we believe it can be applied to both low- and high-density libraries. The proposed method has been titled TnDivA (Transposon Diversity Analysis) and stored as an R package within Github which can be assessed at (https://github.com/gayatri-101/TnDivA).

### Comparison of gene fitness results generated via TnDivA using published comparative tools

To compare and validate the fitness results of genes and the pivotal COG categories generated via TnDivA, firstly, the TnSeq dataset of *N. aromaticivorans* was analyzed through the “resampling” tool for pairwise comparison within the TRANSIT software [75]. This analysis was employed through the BV-BRC platform. Secondly, “two sample analysis” test was performed in TSAS software [76]. Raw .fastq file were used and TRANSIT operated in “resampling” mode to summarize the output file providing information on gene fitness difference between conditions.

In TSAS, mapped reads generated through the Bowtie mapping tool in Sam files format were used as an input to conduct comparative analysis between control and treatment replicates. Provided TSAS codes were run on JAVA runtime using the detailed instructions-to-use on TSAS user guide https://github.com/srimam/TSAS/blob/master/TSAS/V0.2.9/TSAS%20User%20Guide.pdf.

### TnDivA analysis of *Mycobacterium tuberculosis* TnSeq data

TnDivA was used to analyze previously published *Mycobacterium tuberculosis* TnSeq data that was prepared using modified himar1-based transposons [77]. The original library size consisted of approximately 10^5^ independent pooled insertions. Subsequent growth of these libraries was carried out in duplicate for glycerol and triplicate for cholesterol, growing up to 12 generations in each defined media. The insertion density of the replicates was in the range of 40–60%, with mean template-counts ranging from 50 to 90 per TA site [77]. To analyze the fitness difference per gene between conditions, TnDivA was performed on the published data as described above, leveraging all the replicates per condition. The generated effective density was analyzed for significant statistical difference between conditions for every gene using the three different default statistical methods applied in the TnDivA approach, including log_2_-fold change, Cook’s distance and Welch’s t-test at a cutoff of both p ≤ 0.05 and p ≤ 0.01 as described above.

## Results

### Validation of viability during cold stowage and generations of growth in FPAs

**Fig. 2:**
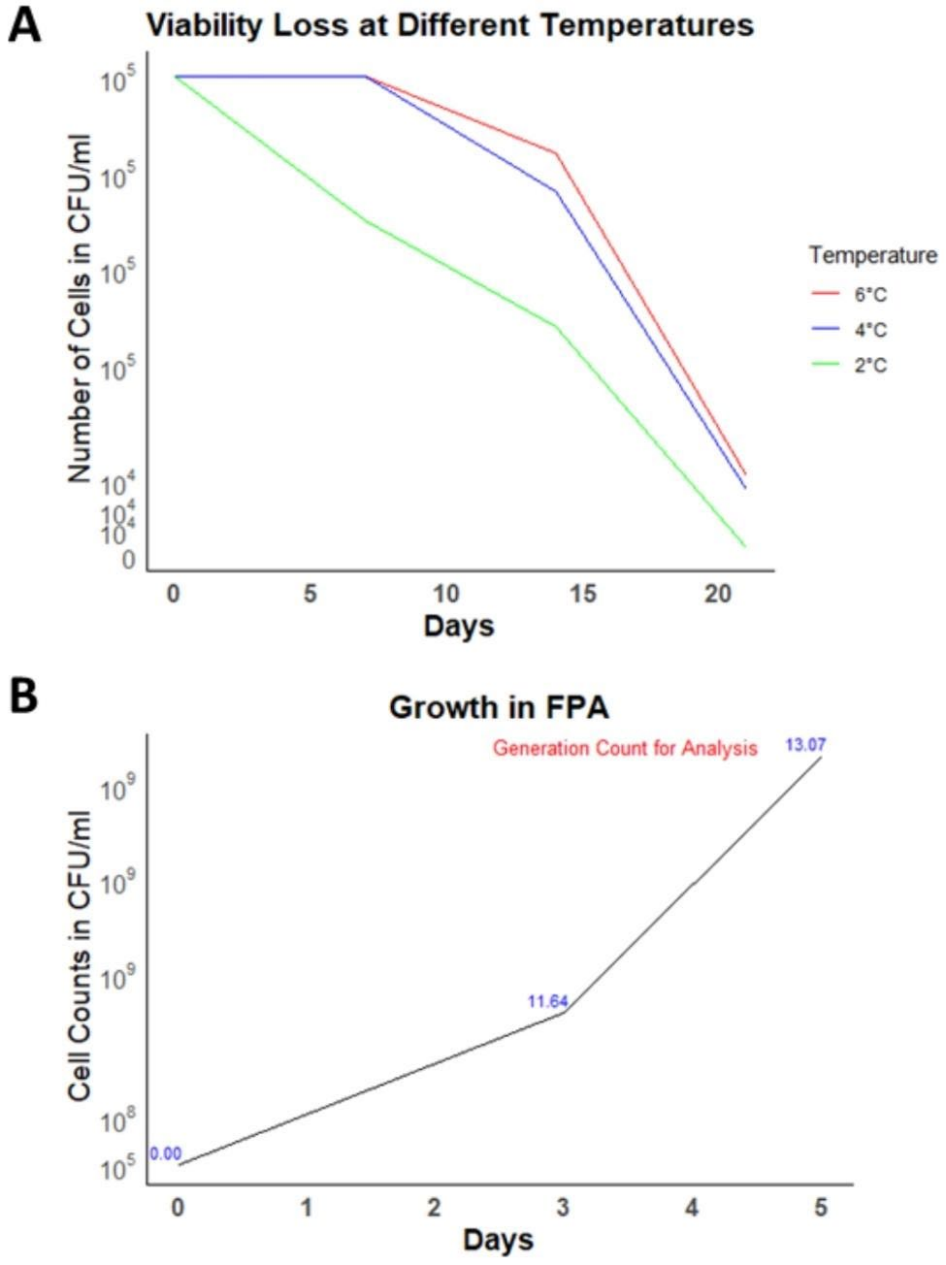
Assessment of viability during cold stowage and growth time in FPAs. (A) *N. aromaticivorans* TnSeq libraries were assessed for viability loss during storage at low temperatures. Cells were resuspended in carbon-deficient 1X M2 salts in FPAs and incubated at 2 ^o^C, 4^ o^C and 6 ^o^C for 21 days. One replicate was removed every 7 days. Cells were serially diluted and plated on PYE agar plates and CFU/ml was calculated. (B) *N. aromaticivorans* TnSeq libraries were cultured inside FPAs and CFU/ml was assessed over 5 days. The numbers in blue indicate number of growth generations at each time point. The number of generations of growth at the time of sample extraction for the analysis has been indicated in red

The cold storage temperature and time to maintain viability of mutant cells were validated by storing and checking for variation in cell count under three different temperatures, 2 °C, 4 ^o^C and 6 °C, for 21 days inside FPAs without shaking. The results indicated that at 2 °C, the cell counts decreased after one week of storage (Fig. 2A). At 6 °C and 4 °C, the cells remained viable for up to 14 days with no change in CFU/ml. However, after 14 days 10-fold reduction in viability was observed. Thus, it was determined that the selected bacteria should be stored for no more than 14 days at temperatures ranging from 4 to 6 °C to maintain the viability before initiating growth. During the experiment, cultures were stored at 4^o^C for 11 days between assembly and activation, well within the 14 day time window.

Previous research showed that a minimum of 5 generations of growth is needed to see fitness effects as differences in transposon representation via TnSeq [59]. However, the higher the number of generations, the higher is the probability of extinction or loss due to random chance, which can artificially affect transposon density [59–61]. In a previous study, the observed differences in transposon representation even at 15 generations of growth were attributed to fitness effect rather than random chance [61]. In our study and prior to spaceflight, growth was monitored inside FPAs to identify an incubation time that achieved between 5 and 12 generations of growth. It was determined that 5 days of incubation achieved ~ 13 generations of growth (Fig. 2B) which was considered suitable for assessing fitness. Furthermore, considering that the difference in the number of generations achieved between 3 and 5 days was minimal, it suggests that the growth had reached saturation and provided sufficient biomass. Thus, both ISS and Ground cultures were incubated for 5 days prior to termination of growth by addition of RNAprotect fixative.

### Variability in transposon inserts and abundance between conditions revealed by TnSeq

Genomic DNA from all the ISS and Ground replicates was extracted and sequencing was performed to map transposon insertions and read count data across the genome. To mitigate factors that could artificially inflate the transposon representation and affect the fitness evaluation, such as insertions in the improperly-annotated start sites at the 5’ end of genes or insertions at the 3’ end that do not disrupt protein function, 20% of the coding sequence from both the 5’ and 3’ ends of each gene was eliminated. The data showing the total read count of transposon insertions per gene and read count of individual inserts within a gene for each sample detected from TnSeq is available in Supplementary Data_S3. These data collectively provided a comprehensive view of the transposon insertions across the genome, gene-specific insertion patterns, and the frequency of insertions within each gene. A summary of the total read counts, unique inserts detected and frequency of inserts as read counts for all ISS and Ground replicates is shown in Table 1.

**Table 1:** Summary of TnSeq results covering 60% of coding region

Replicates/sample	Culture condition	Total reads generated per sample	Unique transposon insertion sites	Read counts of transposon insertion sites	% reads mapped to *N. armaticivorans* DSM 12,444
GH2_5	Ground	6,248,855	1,137,106	3,097,214	99.9%
GH2_6	Ground	6,668,904	1,080,470	3,526,336	99.9%
GH2_7	Ground	6,586,205	870,429	2,208,448	65.2%
GH2_8	Ground	618,156	188,961	225,243	73.6%
ISS2_5	ISS	4,956,899	807,195	2,171,979	84.4%
ISS2_6	ISS	1,021,826	346,678	452,011	86.5%
ISS2_7	ISS	4,076,354	757,246	1,884,491	88.6%
ISS2_8	ISS	3,487,400	712,655	1,558,269	84.4%

As a general trend, the ISS replicates had relatively lower numbers of unique transposon inserts and read counts/frequency as compared to Ground replicates. Because the preservative and freezing process effectively killed the cells, no post-growth assessment of biomass could be performed. Moreover, checking the optical density (OD) would not have yielded accurate results due to cell lysis and the presence of cell debris, as observed through microscopic examination. Therefore, it is not clear if the reduced transposon numbers in the ISS samples were the result of comparatively reduced growth or due to some other reason. In the Ground samples, GH2_8 showed dramatically lower counts as compared to other Ground replicates, with roughly one-tenth the total reads of the others. Similarly, the ISS replicate ISS2_6 showed relatively low read counts than others, despite having same culture conditions and starting with the same number of cells. There is no clear explanation for the abnormally low read counts of select samples.

Upon mapping of the reads to *N. aromaticivorans* genetic elements, the majority of reads of each sample mapped to *N. aromaticivorans*, but unfortunately some reads in several samples mapped to other organisms. Out of total reads detected, the percentage of reads mapped to *N. aromticivorans* was 99% in GH2_5 and GH2_6, 65.2% for GH2_7, and 73.6% for GH2_8 (Table 1). All the ISS samples had between 84.4% and 88.6% reads mapping to *N. aromaticivorans*. All reads that did not map to *N. aromaticivorans* were found to be mapped to the *Agrobacterium tumefaciens* str. C58 and thus considered as contaminated reads.

### Calculation of effective density

While the inability to repeat the experiment introduces limitations, careful data processing and analysis can help mitigate potential biases or confounding factors, and still provide valuable insights from the available data. To ensure a robust and reliable analysis to minimize biases and to extract meaningful information, the data was processed in several steps. Firstly, analysis was restricted only to those replicates with more than 80% of the reads mapping to *N. aromaticivorans*. This resulted in removal of two Ground controls, GH2_7 and GH2_8 from the analysis. Secondly, the low read counts observed in ISS2_6 may make it appear that certain genes have artificially high fitness for the condition as they may seem to not have transposon inserts simply due to the limited read counts. To avoid the potential loss of statistical power due to relatively low read counts, two separate statistical analyses were performed: one including ISS2_6 referred to as AI-1, and one without ISS2_6 referred to as AI-2. This revealed how sensitive the analysis method is by determining how much a low read-count sample influenced the results.

Previously published TnSeq analysis methods have relied on transposon density as a proxy for gene fitness, and have employed a variety of methods to sort high fitness genes from low fitness genes [78–80]. However, many of these methods are built to analyze a single TnSeq library and are not suited for comparing TnSeq libraries, and few of them are capable of leveraging sample replicates. Even the methods that can use replicates have primarily focused on quantifying changes in read counts as a measure of fitness. To accurately assess the fitness differences between conditions, it is crucial to consider both the variation in overall transposon diversity and the distribution of read counts within each gene across conditions. This combination of factors has not been adequately captured by previous analytical techniques. Therefore, it was necessary to devise a novel data analysis method that offers a more comprehensive approach.

**Fig. 3:**
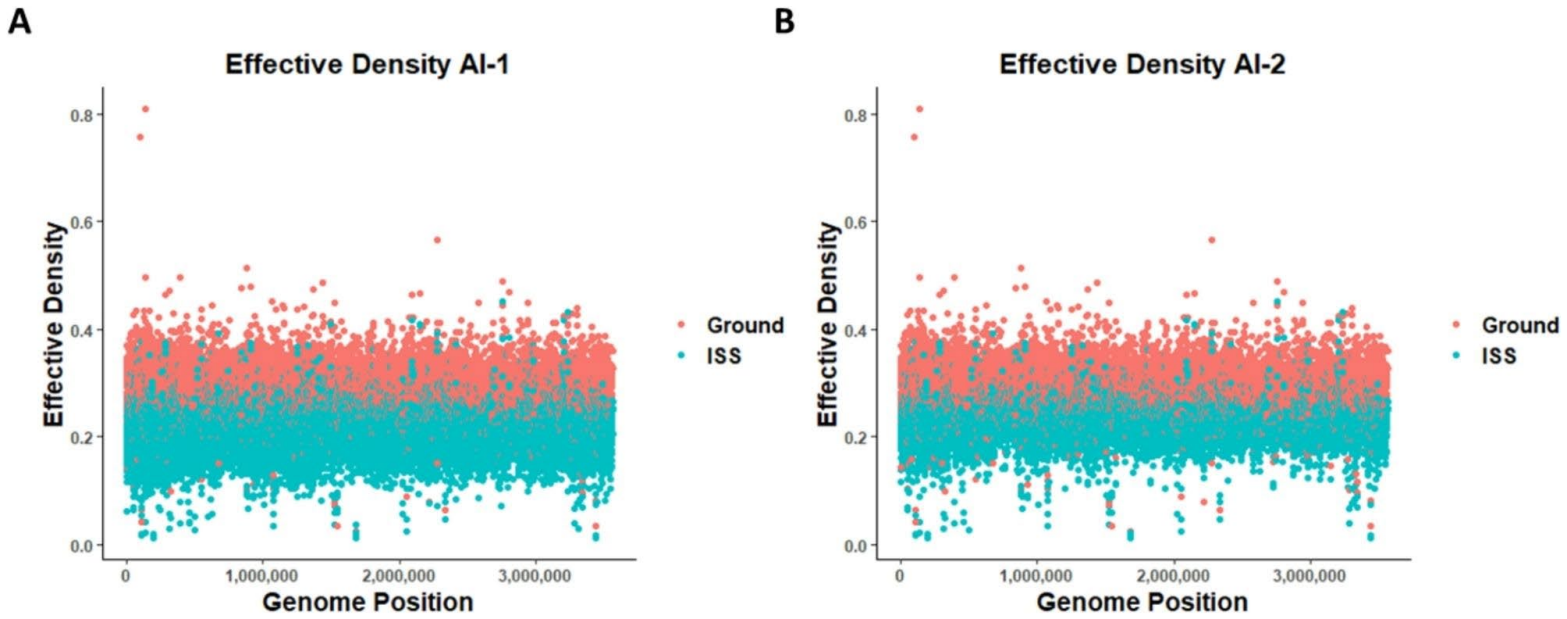
Scatterplot of average effective density for Ground and ISS samples. Average effective density was calculated for each from Ground (orange) and ISS (blue) sample for AI-1 (A) and AI-2 (B) data sets. The average effective densities were then plotted by genome position. ISS samples had consistently lower average effective densities

The statistical comparison of different ecological community compositions using the Shannon diversity index has been performed by ecologists for decades. Here we apply the same approach to a molecular context to calculate the diversity of transposons per gene. The fitness differences between conditions were determined by taking into account both the number of individual unique transposon insertion locations in a gene, referred to as unique inserts, and the number of times each insert was sequenced, collectively considered as frequency or read count data (Supplementary Data_S3). The Shannon Diversity Index is particularly useful in this context as it is a single numerical metric that takes into account both of these factors. However, direct comparison of the index values is unreliable as they lack a doubling property [68]. To overcome this, the exponent of the Shannon Index was calculated to determine the effective number of transposon sites that allows for intuitive and meaningful comparisons between conditions. Effective density better represents transposon diversity while mitigating anomalies caused by aberrant highly abundant transposons. We refer to the use of modified Shannon diversity indices of transposon density to determine gene fitness as Transposon Diversity Analysis, or TnDivA. The effective density per gene was subsequently calculated using this TnDivA methodology for both AI-1 and AI-2 (Supplementary Data_S4). As a general trend, lower effective transposon density values were observed for ISS replicates as compared to Ground. To compare the effective densities between conditions, a distribution plot of effective density was plotted for both AI-1 and AI-2 separately, shown in Fig. 3. There is a clear demarcation between the conditions indicating the difference in effective density between Ground and ISS replicates for all the genes. Most of the effective densities for Ground replicates was distributed at > 0.3 range, with a few relatively higher effective densities in the range of 0.8 and a few distributed at a lower range of < 0.1. For ISS replicates, effective densities were mostly in the range of 0-0.3 with a few genes at around 0.4. The difference in transposon diversity distribution was seen to be more pronounced in AI-1 with less overlapping genes in between, suggesting that the low read counts of ISS2_6 could influence downstream analysis.

### Comparisons of statistical methods to identify genes of potential significance in microgravity

**Fig. 4:**
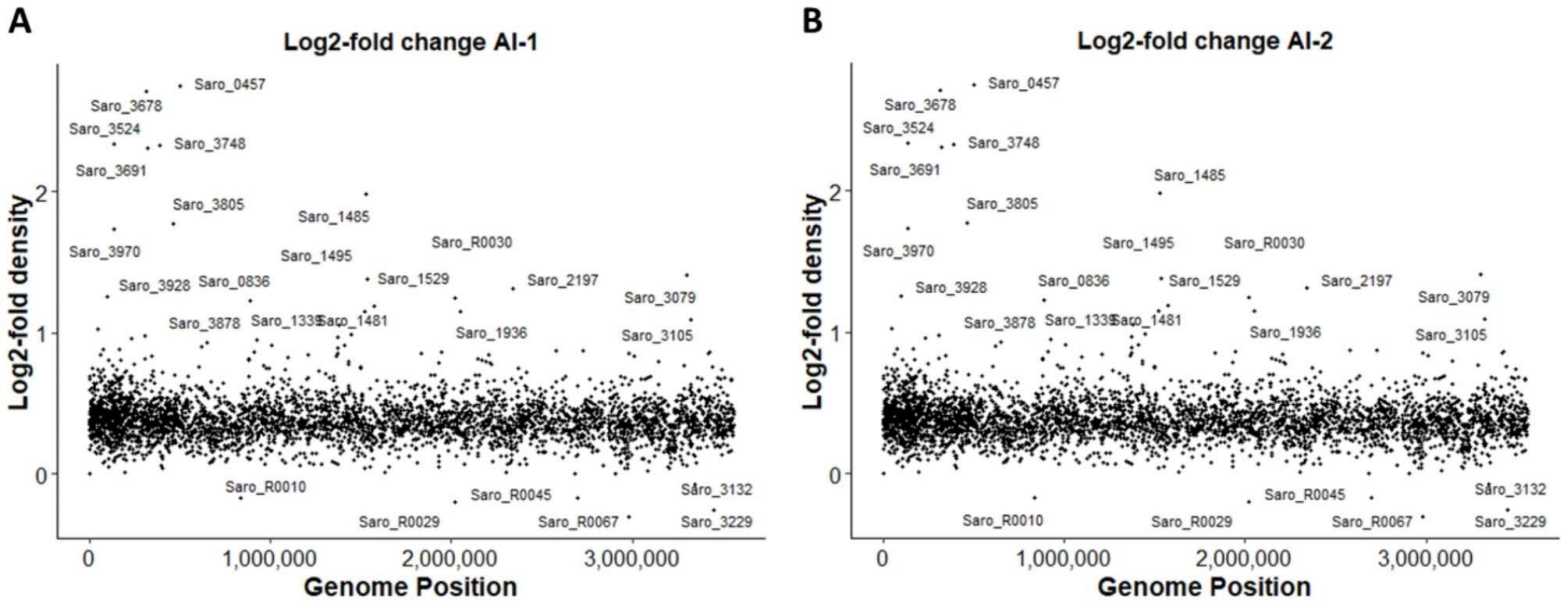
Scatterplot of log2-fold change ratio of average effective densities. For each gene, the average effective density of Ground samples was divided by the average effective density of ISS samples and log_2_ transformed. This was performed for the AI-1 (A) and AI-2 (B) data sets. This data was then plotted by genome position. Genes with ratios above 1 have 2-fold higher effective density in Ground replicates compared to ISS replicates, suggesting transposon insertions in these genes caused reduced fitness in microgravity. The displayed scattered points indicate the 22 outlier genes in the AI-1 and 20 outlier genes in the AI-2.

To identify genes that exhibit significant differences in effective density between ISS and Ground samples, a log_2_-fold change method was applied. The effective density values of each gene were averaged across replicates for each condition. The average Ground effective density was divided by the average ISS effective density for every gene, and the resulting ratio was then log_2_-transformed, quantifying the fold change in effective density per gene (Supplementary Data_S5). The transformed values for each gene were plotted as shown in Fig. 4, where positive values indicated higher effective density in the Ground samples, and negative values indicated higher effective density in the ISS samples. A large proportion of genes across the genome were clustered within the range of 0 to 1. This is likely caused by the globally lower effective density values in the ISS data. A cutoff of values > 1 was used to identify genes with high fitness under microgravity. These values indicate a two-fold higher ratio of effective density in Ground samples compared to ISS samples, suggesting the transposon diversity in those genes was much higher on Ground than aboard the ISS and thus mutations in those genes have a fitness cost in microgravity. In the AI-1 analysis, 22 genes were identified as significant (Supplementary Data_S5), while 20 genes were considered significant in AI-2 (Supplementary Data_S5). Out of all the genes identified, 18 genes were identified to be common to both analyses (Supplementary Data_S6). Therefore, in the log_2_-fold change method, inclusion of the low read ISS sample had little impact on identification of significant genes.

**Fig. 5:**
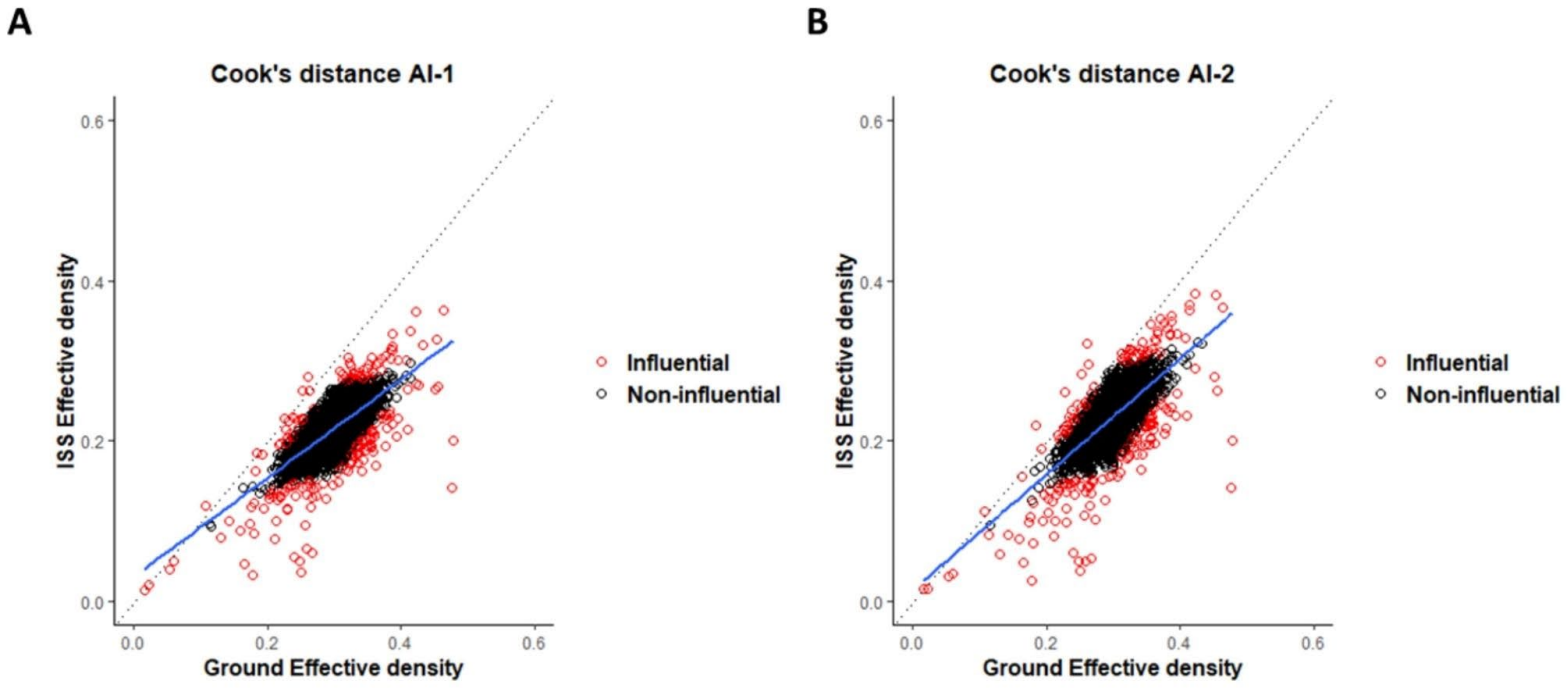
Scatterplot of mean effective density data of Ground vs. ISS samples with linear regression line. Average effective densities from the AI-1 (A) and AI-2 (B) data sets were used to calculate linear regression models. Dashed lines indicate the ideal model where no difference between conditions is observed, while the blue lines represent the model fit to the effective density data. Disproportionate distribution of genes along the fitted line was observed due to 200 influential genes in AI-1 and 199 influential genes in AI-2, indicated in red, which had a significant impact on the regression model. These influential genes pulled the fitted line towards the Ground genes

As an alternative method to identify genes of interest, a linear regression model was generated to identify outliers or influential genes based on their Cook’s distance measures, as shown in Fig. 5. In the AI-1 analysis, 200 genes were found to have Cook’s distance values more the 4/n (Adjusted R^2^: 0.5242, p-value: < 2.2e-16) (Supplementary Data_S7). These included all 22 genes identified as potentially significant genes through the log_2_ transformation analysis. Similarly, in AI-2, 199 genes (Adjusted R^2^: 0.5242, p-value: < 2.2e-16) were identified as influential genes (Supplementary Data_S7), with all 20 genes in common using the log_2_ transformation analysis. From both AI-1 and AI-2, a set of 159 genes were identified as common influential genes (Supplementary Data_S8). This data set included all 18 genes in common between log_2_ transformation in AI-1 and AI-2 analysis. The fact that these common genes were consistently identified across multiple analysis methods indicates their potential importance and suggests that they should not be overlooked. Furthermore, the presence of significant overlap in genes between AI-1 and AI-2 analyses provide additional evidence that the low read counts did not have a substantial impact on the statistical power of the linear regression analysis. This strengthens the confidence in the findings that the identified genes are indeed relevant to understanding the fitness differences in microgravity conditions.

Another analytical method, one specifically afforded by using experimental replicates, is statistical comparisons using Welch’s t-test on each individual gene. Using this method, at a p ≤ 0.05, 1096 genes were identified as having statistically lower effective densities in the ISS condition in AI-1 (Supplementary Data_S9). This represents approximately one-fourth of the total genes of this organism and suggests that a p ≤ 0.05 is not stringent enough a cutoff. When a more stringent cutoff of p ≤ 0.01 was applied, the number of significant genes decreased to 152 (Supplementary Data_S9). This reduction in significant genes suggests that the larger number at p ≤ 0.05 may be influenced by the global lower effective densities observed in the ISS samples. For the AI-2 data set, at p ≤ 0.05, 652 genes exhibited a statistically significant difference in mean effective density (Supplementary Data_S10). When a more stringent cutoff of p ≤ 0.01 was applied, the number of significant genes decreased to 199 (Supplementary Data_S10). Furthermore, when considering genes identified as significant at the cutoff of p ≤ 0.01 for both AI-1 and AI-2 analysis, a total of 69 genes were found to be common (Supplementary Data_S11). This is less than half the genes in either data set, which is much lower than the consensus genes of the linear regression analysis (common genes comprised approximately three-quarters of each data set). These results suggest that Welch’s t-test as an analytical method is much more sensitive to an anomalously low read count sample.

The genes identified using Welch’s t-test were examined to see if they were also outlier genes identified through the Cook’s distance and log_2_-transformed data. Clearly, genes that were significant across all the analyzed matrices and statistical tests must be crucial to survival in microgravity. Out of 199 influential genes detected in AI-2, as identified through Cook’s distance, only 20 genes were found to be statistically significant at a cutoff of p ≤ 0.05, and none identified through log_2_-transformation. Using p ≤ 0.01, only 9 influential genes identified by Cook’s distance were found to have significant differences in their effective densities between conditions, and again none were found in the log_2_-transformation analysis. These results show a shocking lack of consensus between the analysis methods. All three methods employed in this study utilize different statistical approaches. In our analysis, the interpretation of log_2_-fold change lacked statistical support when compared between the analytical methods. Log_2_-fold change and Cook’s distance appeared to be more robust in identifying significant genes capturing a wide range of magnitude differences, specifically log_2_-fold change being more stringent. On the other hand, Welch’s t-test, although allowing for consideration of genes with small fold-changes as statistically significant, did not identify all genes with higher magnitude differences. Consequently, there was limited overlap between the significant genes identified by Welch’s t-test and the other methods. Welch’s t-test exhibited a moderate association with Cook’s distance and a weak association with log_2_-fold change. Importantly, no single method demonstrated clear superiority over the others. Considering both the p-values in Welch’s t-test and log_2_-fold change as well as identifying outliers through Cook’s distance proved crucial. Each approach contributed to the identification of a subset of important genes associated with microgravity. However, targeted experimental validation is necessary to evaluate the confidence and reliability of these findings.

### COG category and functional analysis of genes with high fitness in microgravity

Genes identified as significant from the various analytical techniques were categorized based on predicted COG function to determine if certain cellular processes were more affected by growth in microgravity than others. COG categories were examined for all the genes detected through all three statistical methods, which increased confidence in determining whether a consistent pattern in the categories emerged across different statistical methods. The analysis of both log_2_-fold change and linear regression using Cook’s distance had minimal difference when incorporating low read replicates in AI-1 compared to AI-2 dataset. Additionally, reduced variability between replicates in AI-2 dataset also led to decreased risk of type I error, as observed in the reduction of significant genes from AI-1 to AI-2. Given that, our downstream analysis will focus exclusively on the AI-2 dataset. However, details of the analysis for the AI-1 dataset are provided in the supplementary files (Supplementary_Data_S12).

**Fig. 6:**
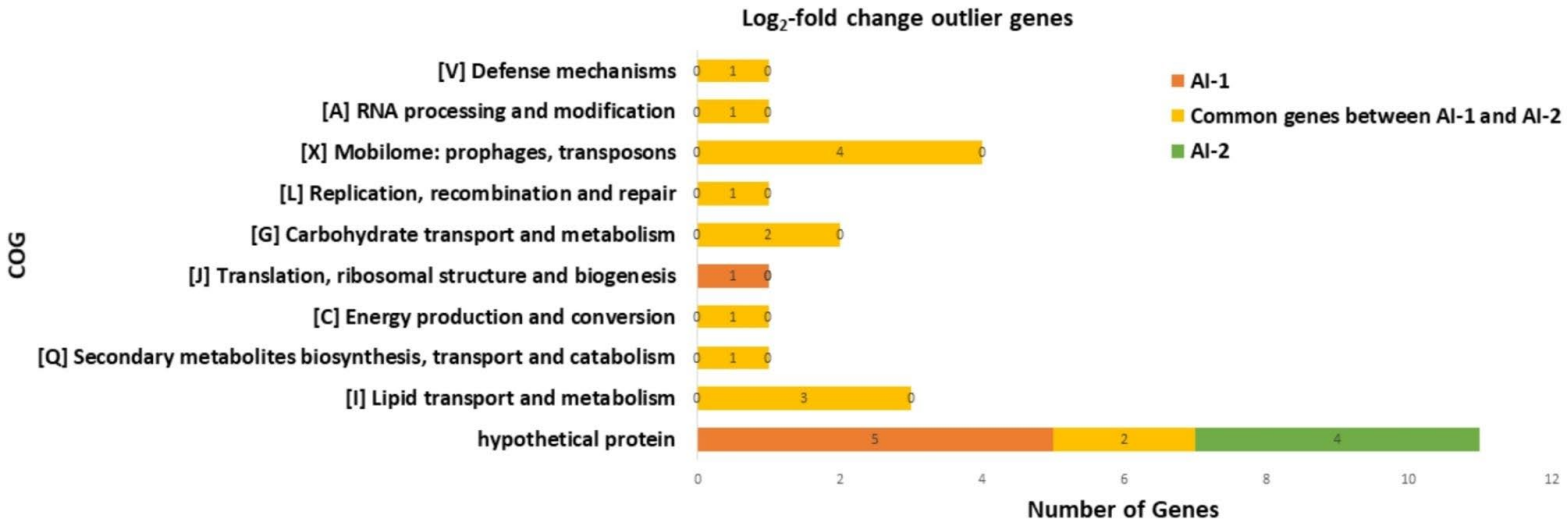
COG category prediction of genes identified as significant by log_2_-fold change. Genes from the AI-1 (orange) and AI-2 (green) or both (yellow) data sets that had log_2_-fold change values of 1 or greater were assessed for COG category prediction. In the case where a given gene had more than one COG prediction, all predictions were used, meaning that there are more predictions than the total number of genes. Total number of genes for both data sets (AI-1 and AI-2) is the sum of number of genes in each data set and the common genes

It is important to note that some genes have multiple COG predictions, resulting in the total number of predictions being more than the total number of genes. Additionally, the presence of large number of hypothetical genes can be attributed to the fact that approximately 38.48% of the genome is annotated for hypothetical, unknown function, and general function prediction genes in this organism. A summary of COG category prediction from genes identified as significant from log_2_-fold change is presented in Fig. 6. Among the 20 significant genes identified as significant/outlier (> 1 log_2_-fold) in AI-2 dataset, 6 genes were categorized as hypothetical genes. The functional gene categories identified were lipid metabolism and transport with 3 genes, carbohydrate transport and metabolism with 2 genes and 1 gene each for secondary metabolite biosynthesis and energy production.

**Fig. 7:**
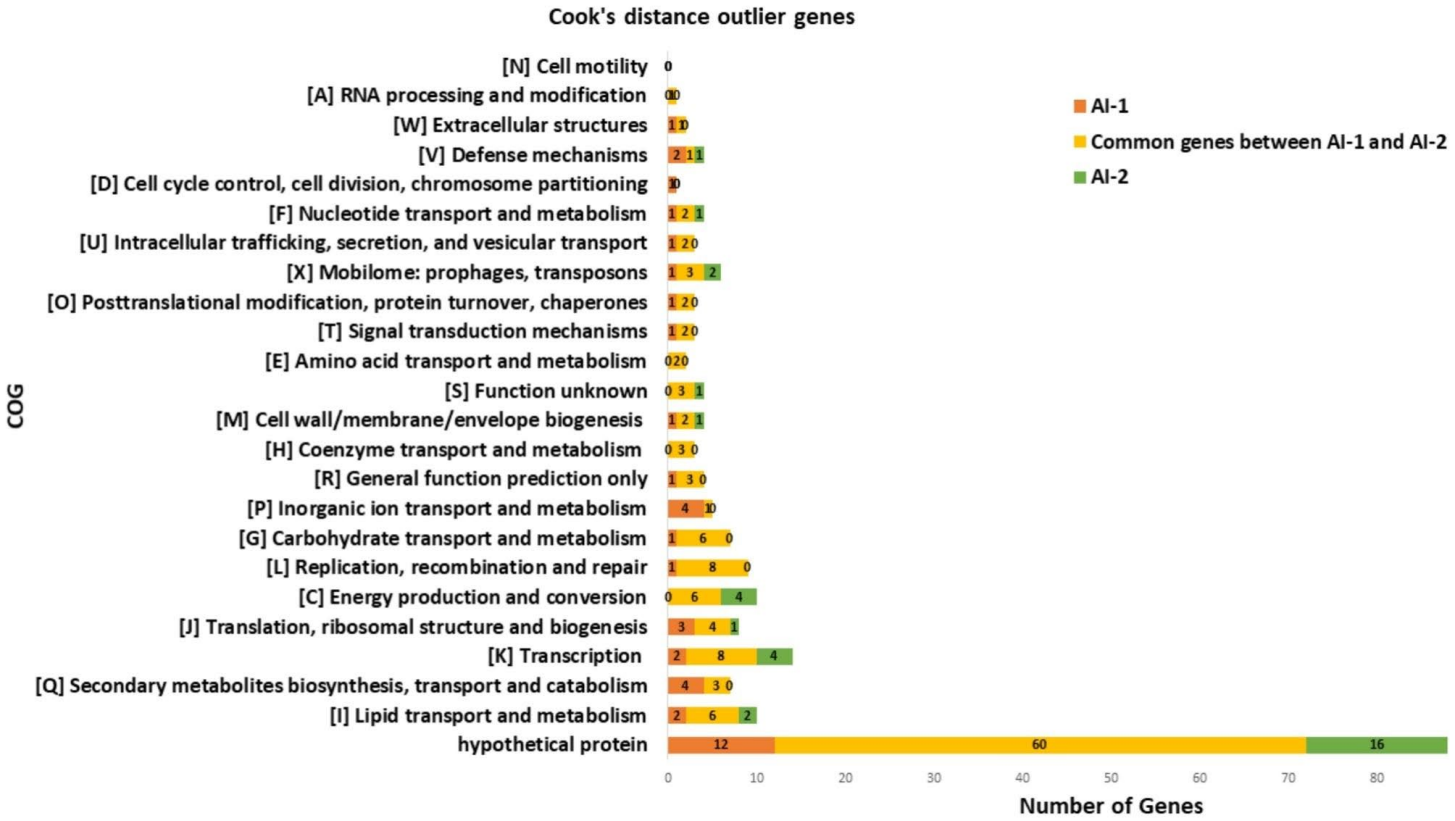
COG category prediction of genes identified as significant by Cook’s distance. Genes identified as influential by Cook’s distance from the AI-1 (orange) and AI-2 (green) or both (yellow) data sets were assessed for COG category prediction. In the case where a given gene had more than one COG prediction, all predictions were used, meaning that there are more predictions than the total number of genes. Total number of genes for both data sets (AI-1 and AI-2) is the sum of number of genes in each data set and the common genes

When the genes identified as significant/influential through Cook’s distance were categorized for predicted COG function (Fig. 7), similar functional categories were found as the log_2_-fold change significant genes; this is unsurprising given the significant overlap in the results (see previous section). In AI-2, out of 199 statistically significant influential genes, 76 were hypothetical genes, 3 were general function prediction genes and 4 were unknown function genes. Likewise, the most represented functional gene categories were lipid metabolism and transport with 8 genes, transcription with 12 genes, carbohydrate transport and metabolism with 6 genes, and replication, repair and recombination with 8 genes, and energy production and conversion with 10 genes. Only 3 genes were detected to be associated to secondary metabolite biosynthesis.

**Fig. 8:**
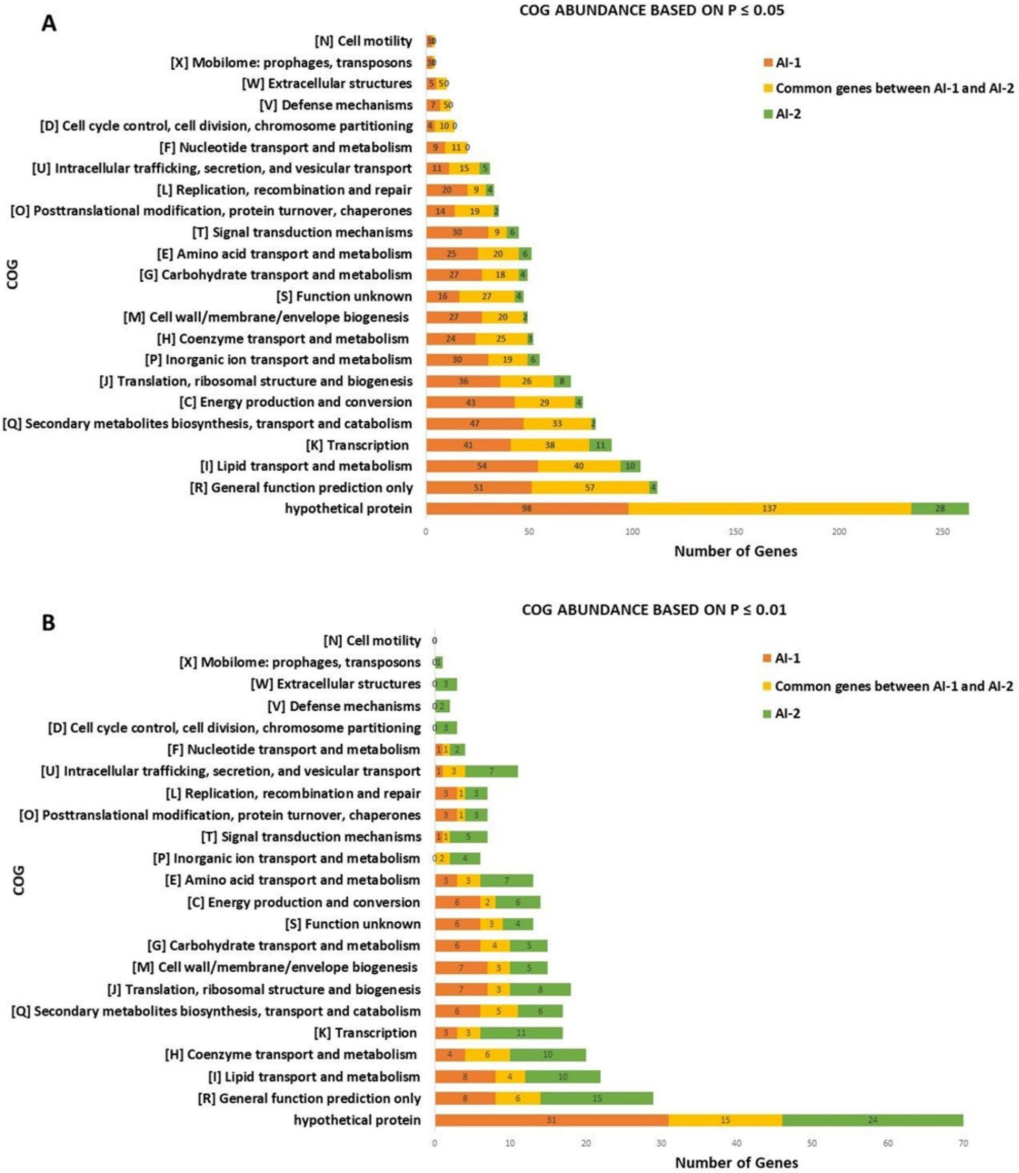
COG category prediction of genes identified as significant by Welch’s t-test. Genes identified as significant by Welch’s t-test from the AI-1 (orange), AI-2 (green), or both (yellow) data sets, at p ≤ 0.05 (A) and p ≤ 0.01 (B) were assessed for predicted function by COG category. Total number of genes for both data sets (AI-1 and AI-2) is the sum of number of genes in each data set and the common genes. In the case where a given gene had more than one COG prediction, all predictions were used, meaning that there are more predictions than the total number of genes

While the log_2_-fold change significant genes and Cook’s distance significant genes had extensive overlap, the overlap between these genes and those found significant by Welch’s t-test was considerably less (see previous section). The Welch’s t-test significant genes were analyzed for predicted COG function to determine if predicted COG function was similarly distinct. In AI-2 data set, 652 genes were significant at the p ≤ 0.05 cutoff (Fig. 8A). The most abundant COG category was hypothetical genes with 165 genes, 31 genes had unknown function, and 61 were categorized as general function prediction. The most represented functional gene categories again included 50 genes associated with lipid transport and metabolism, 35 genes associated with secondary metabolite biosynthesis, transport, and catabolism, and 49 genes involved in transcription (Fig. 8A).

**Fig. 9:**
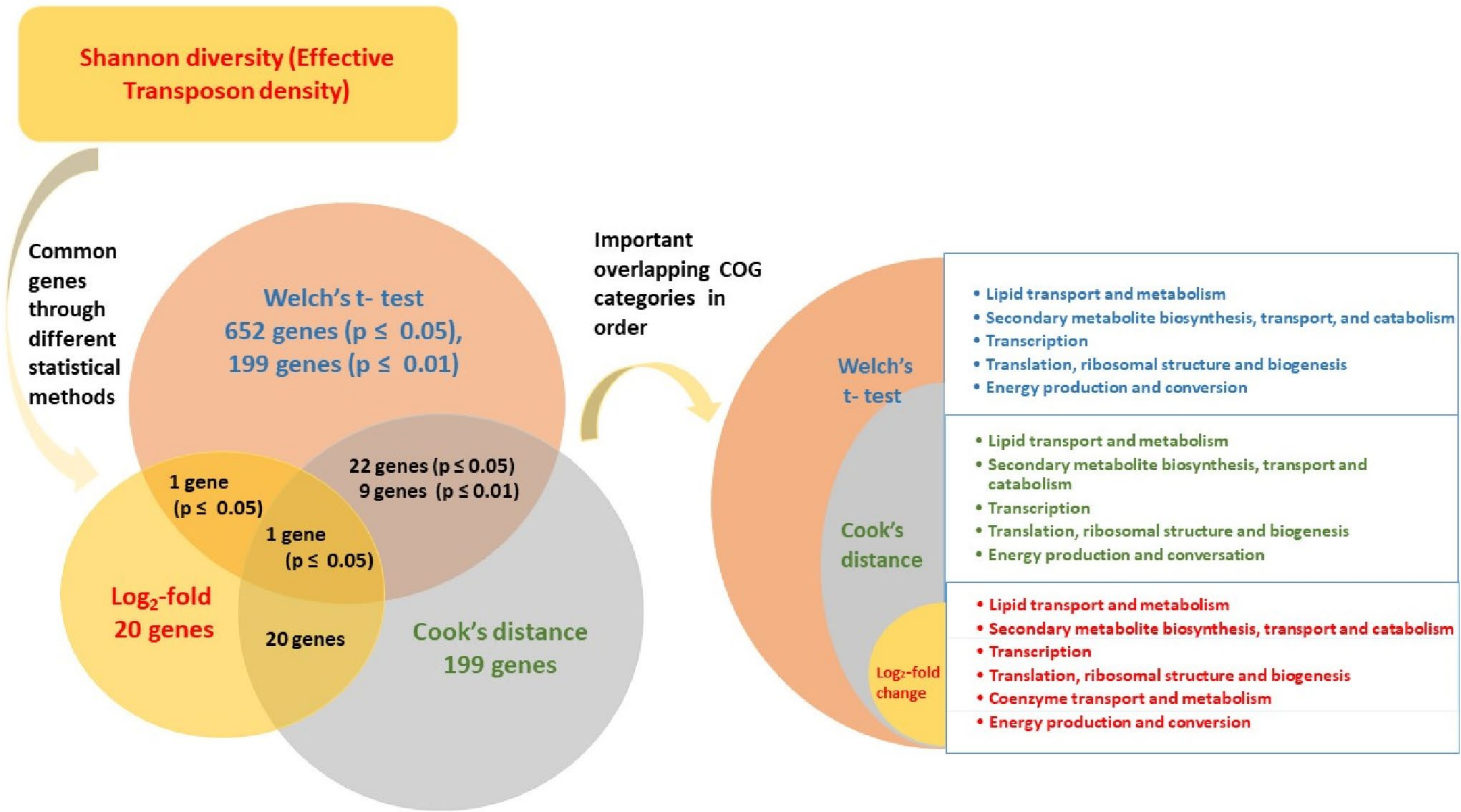
Summary of significant genes from three analytical methods. Diagram summarizing the numbers and overlap of important genes identified across three analysis methods and COG categories associated with genes identified as significant to growth in microgravity, listed based on their abundance

While changing the statistical cutoff to the more stringent p ≤ 0.01 reduced the total number of significant genes, a similar pattern in the abundance of genes based on their function was observed (Fig. 8B). In AI-2, out of 199 genes identified as statistically significant, 39 genes were hypothetical, 7 genes had unknown function, and 21 genes were assigned as having a general function prediction. Among the genes with known function, the most represented groups were 14 genes each related to lipid transport and metabolism and transcription, 16 in coenzyme transport and metabolism, 14 in transcription, 11 genes involved in secondary metabolite biosynthesis, transport, and catabolism, and 11 genes related to translation, ribosomal structure, and biogenesis. These results demonstrate congruence between the AI-1 and AI-2 data sets at the different statistical cutoffs, suggesting this analysis method was robust at the functional prediction level (Fig. 8B). More importantly, the results are also congruent with the other analytical methods with overlapping significant genes at the predicted functional level as well (Fig. 9). The more prevalent functional categories from the log_2_-fold change and Cook’s distance analyses were lipid transport and metabolism, secondary metabolites, transcription, translation, and carbohydrate metabolism; these same categories were prevalent in the Welch’s t-test results in AI-2 at the various cutoffs. Therefore, while there may be relatively less overlap in the individual genes when comparing analytical methods, there is large overlap in COG category predictions, suggesting these biological processes are the ones most impacted by culturing in microgravity (Fig. 9).

To further examine functions important for growth in microgravity, the biological roles of some of the genes consistent between analytical methods were analyzed in greater detail. In the AI-2 dataset, no gene was found significant in all three analytical techniques. However, in the AI-1 dataset, one significant gene, *saro_1339*, was found common for log_2_-fold change, Cook’s distance and Welch’s t-test at p ≤ 0.05. This gene, associated with lipid metabolism and transport and encodes an acyl carrier protein which is believed to be involved in the fatty acid biosynthetic pathway. It transports the growing fatty acid chain between enzyme domains of fatty acid synthase. Genes under this category are highly abundant in all analytical methods and datasets.

When the relatively stringent log_2_-fold change analysis was removed from consideration, 20 outlier genes from Cook’s distance were found in the AI-2 dataset at p ≤ 0.05 (Table 2). Of these 20, 11 were classified as hypothetical genes, 3 were involved in lipid metabolism and transport, 2 were associated with secondary metabolite biosynthesis, transport, and catabolism, 2 was transcription-related, and 1 gene was a nuclease.

**Table 2:** Summary of 20 common genes identify through Cook’s distance and Welch’s t-test

Locus Tag	ED (G)	ED (ISS)	Influential (> 4/n Cook’s distance)	Welch’s t. test (p ≤ 0.05)	low fitness	Gene Product	COG	Gene functions
Saro_0557	0.518163423	0.286886804	Yes	0.00213106	ISS	nuclease (SNase-like)	nuclease (SNase-like)	Hydrolase activity, a on ester bonds
Saro_1063	0.48046069	0.278912913	Yes	0.008205582	ISS	hypothetical protein	hypothetical protein	
Saro_1339	0.454544094	0.234475765	Yes	0.029067257	ISS	acyl carrier protein	[I] Lipid transport and metabolism [Q] Secondary metabolites biosynthesis, transport and catabolism	Transports the growing fatty acid chain between enzyme domains of fatty acid synthase (FAS) during biosynthesis
Saro_1351	0.516126475	0.394114113	Yes	0.018421515	ISS	transcriptional regulator, AsnC family	[K] Transcription	Amino acid metabolism, regulation of transport processes or cell morphogenesis.
Saro_1612	0.51261362	0.337969777	Yes	0.009293063	ISS	hypothetical protein	hypothetical protein	
Saro_2006	0.513531587	0.278627056	Yes	0.005967643	ISS	hypothetical protein	hypothetical protein	
Saro_2014	0.537626755	0.343486828	Yes	0.038028149	ISS	heat shock protein Hsp20	[O] Posttranslational modification, protein turnover, chaperones	Molecular chaperone IbpA, HSP20 family
Saro_2033	0.455425818	0.236599201	Yes	0.029024472	ISS	hypothetical protein	hypothetical protein	
Saro_2246	0.504140769	0.293825409	Yes	0.018279786	ISS	hypothetical protein	hypothetical protein	
Saro_2725	0.472698515	0.251977191	Yes	0.004722643	ISS	hypothetical protein	hypothetical protein	
Saro_2874	0.500877253	0.292535145	Yes	0.009886839	ISS	Twin-arginine translocation pathway signal	Twin-arginine translocation pathway signal	Export of folded proteins across the cytoplasmic membrane of bacteria.
Saro_3006	0.535912608	0.464719652	Yes	0.041766568	ISS	hypothetical protein	hypothetical protein	
Saro_3077	0.531596141	0.354834984	Yes	0.031025729	ISS	hypothetical protein	hypothetical protein	
Saro_3890	0.520015364	0.428781833	Yes	0.033590134	ISS	fatty acid desaturase	[I] Lipid transport and metabolism	Catalyze the biosynthesis of monounsaturated fatty acids (MUFAs) and polyunsaturated fatty acids (PUFAs).
Saro_3949	0.530724879	0.416706415	Yes	0.010790725	ISS	hypothetical protein	hypothetical protein	
Saro_3426	0.528715758	0.320315505	Yes	0.0171952	ISS	Glyoxalase/bleomycin resistance protein/dioxygenase	[Q] Secondary metabolites biosynthesis, transport and catabolism	Catechol 2,3-dioxygenase or other lactoylglutathione lyase family enzyme
Saro_3444	0.482016209	0.257009743	Yes	0.020568395	ISS	hypothetical protein	hypothetical protein	
Saro_3600	0.440391595	0.261442673	Yes	0.00208821	ISS	short-chain dehydrogenase/reductase SDR	[I] Lipid transport and metabolism	NAD(P)(H)-dependent oxidoreductases involved in metabolism of a lipids and lipid A modification
Saro_3746	0.528729413	0.418257138	Yes	0.021791329	ISS	hypothetical protein	hypothetical protein	
Saro_3770	0.429515955	0.238187673	Yes	0.002242064	ISS	regulatory protein, LuxR	transcription regulatory protein, LuxR	Coordinates the expression of genes, including encoding for virulence factors and antibiotics biosynthesis

*ED: Effective density, *G: Ground, *ISS: International Space Station

On examining the function of common genes, we found that genes categorized under lipid transport and metabolism were often associated with fatty acid biosynthesis, modification, and metabolism. Enzymes/proteins such as acyl carrier proteins, a fatty acid desaturase, and a short-chain dehydrogenase/reductase (SDRs) encoded by *saro_1339*, *saro_3890* and *saro_3600* genes respectively were important for microgravity. The gene found to be involved in secondary metabolites biosynthesis, transport, and catabolism category, *saro_3426* encodes lactoylglutathione lyase family enzyme (glyoxalase/bleomycin resistance protein/dioxygenase catechol 2,3-dioxygenase). These enzymes have distinct roles including participating in the catabolism of aromatic hydrocarbons, detoxifying harmful byproducts, and facilitating their transport. Given that aromatic compounds were not specifically provided in the growth medium, one potential explanation for these results in the detoxification aspect of this metabolism, which may be important under the stressful microgravity conditions. A third functional category found important in this analysis are genes involved in transcription. When analyzed, the important genes were typically not involved in the process of transcription, but belong to transcriptional regulator families including AsnC (encoded by *saro_1351*) and LuxR (encoded by *saro_3770*). These results suggest that growth in microgravity leads to large transcriptional responses in the cell.

Upon further examination of the larger datasets using all three statistical methods, genes encoding enzymes involved in fatty acid biosynthesis were consistently found. These genes included *saro_1903*, which encodes acetyl-CoA carboxylase and was detected through Welch’s t-test, and acyl-CoA dehydrogenase (encoded by *saro_3691* and *saro_3748*), were identified using both log_2_-fold change and Cook’s distance. These results suggest that fatty acid composition may be altered in response to growth in microgravity. Further genes associated with secondary metabolism were also identified, including *saro_0539*, encoding 2-chlorobenzoate 1,2-dioxygenase, and *saro_0904*, encoding the phenylacetic acid degradation-related protein. Both were detected via Welch’s t-test. The gene *saro_1228*, a member of the thioesterase superfamily, was identified using Cook’s distance. More in-depth analysis of gene function revealed even more transcriptional regulators. LysR regulators, encoded by *saro_0541* and *saro_1678*, were identified through Welch’s t-test and Cook’s distance. Welch’s t-test additionally found members of TetR, LacI, AraC, and MarR regulator families (encoded by *saro_0706, saro_0755, saro_0974*, and *saro_1073* respectively). The consistent detection of genes with functions in maintaining bacterial growth and for protection against stressful conditions strongly suggests that the low-shear environment of microgravity is a potent stressor for bacterial cells.

### Comparison of TnDivA with two published comparative TnSeq analysis tools: TRANSIT and TSAS

To compare the TnDivA method with previously published TnSeq analysis tools with comparative capabilities, the TnSeq data generated here was analyzed using TRANSIT and TSAS software. The data was first analyzed with TRANSIT. TRANSIT utilizes a modified permutation test on the difference of the mean counts between the two conditions for each gene. The quantitative changes in insertion counts reflect apparent fitness change for mutants. A resampling distribution is created by shuffling the observed counts, allowing for the assessment of differences expected by chance under the null hypothesis that conditions are not different. As an output, p-value is calculated based on how often reshuffled samples produce a more extreme difference than the actual data. Conditionally essential genes exhibit a significant difference not attributable to chance, and the method can identify genes with reduced fitness per conditions. Out of the total 4054 genes analyzed, none were identified as conditionally essential or having difference in fitness (adjusted p-value ≤ 0.05) between conditions (Supplementary_Data_S13). To investigate whether the absence of differences stemmed from statistically nonsignificant variances in mean insertion counts between conditions, we performed an ‘Essentiality’ check for each condition using either the gumbel or Tn5gap tool within TRANSIT on the BV-BRC platform. These analyses focus on genes with statistically significant gaps or consecutive sequences of TA sites lacking insertions i.e., empty sites with counts of 0 [81], and can be executed across multiple replicates. The output revealed that no genes were essential under either Ground or ISS conditions (adjusted p-value < 0.05) (Supplementary_Data_S14). Therefore TRANSIT analysis provided no useful output for comparison.

TSAS analysis assesses differential gene fitness between datasets by calculating the average reads per unique insert for each gene, and then calculates the average of the replicates for each gene. These averages are compared between conditions, thereby enabling the calculation of a fold change based on differential abundance of average reads. It determines the statistical significance by calculating associated adjusted p-values corrected using Benjamini Hochberg (BH) method. Genes with significantly fewer reads in the treatment compared to the control are deemed conditionally essential. TSAS also provides a reference for the expected number of insertions and reads in genes under a control condition, eliminating the need for assumptions about the data distribution. However, TnDivA differs from TSAS because it not only integrates both unique insertions and read counts (abundance) but also addresses the issue of over- or under-abundance among unique inserts per gene, such as cases where a few transposon inserts have an abnormally high number of reads, while in others read frequency is more evenly distributed. This consideration prevents potential misinterpretations of the variation in transposon diversity providing a more comprehensive understanding of fitness. TSAS analysis detected 239 genes as conditionally essential for growth aboard the ISS (log_2_-fold change < -1 and adjusted p-value < 0.05) (Supplementary_Data_S15). Comparing TSAS identified conditionally essential genes with TnDivA identified genes from AI-2 using Welch’s t-test, 45 common genes were identified (Supplementary_Data_S16). These common genes included 7 genes that were significant among Cook’s distance and Welch’s t-test for AI-2 analysis, specifically 2 genes that are associated with lipid biosynthesis (*saro_1339* and *saro_3600*), and one transcription regulator associated gene *(saro_3770*), along with other hypothetical genes. Within the common set of 45 genes, the most abundant category consisted of 16 hypothetical genes, followed by 8 genes involved in lipid transport and metabolism, 4 genes associated with transcription, 6 genes involved in secondary metabolite biosynthesis, transport, and catabolism, 2 genes related to translation, and 1 gene associated with energy production and conversion (Supplementary_Data_S16).

### Reanalysis of published TnSeq data using TnDivA

To further compare TnDivA analysis with other comparison tools, TnDivA analysis was performed on a previously published TnSeq data set of *Mycobacterium tuberculosis* H37rv grown on glycerol- or cholesterol-supplemented media [77]. Observations of effective density in cholesterol and glycerol replicates revealed some genes with higher density in cholesterol and others in glycerol, indicating varying fitness of genes across growth conditions (Supplementary_Data_S17). In the TnDivA analysis, the different statistical methods identified similar numbers of genes. A cutoff of > 1 log_2_-fold change revealed 182 genes to have twice the effective density in glycerol as compared to cholesterol growth conditions. Cook’s distance identified 196 genes as outliers or influential genes (adjusted R^2^: 0.678, p-value: < 2.2e-16). Whereas Welch’s t-test revealed 184 genes having a statistically significant difference at p ≤ 0.05, and 40 genes at p ≤ 0.01 (Supplementary_Data_S17).

Genes identified through log_2_-fold change were compared with those identified through Welch’s t- test. Out of 182 genes identified through log_2_-fold change, only 59 genes were statistically significant at a cutoff of p ≤ 0.05, with 21 genes at p ≤ 0.01. These genes showed lower effective density in cholesterol growth media, indicating low fitness in that condition. Similarly, out of 197 influential genes detected through Cook’s distance, only 27 genes were statistically significant at p ≤ 0.05, and 7 genes at p ≤ 0.01. The effective density of most genes was also higher in glycerol growth conditions, with only two hypothetical genes having higher effective density under cholesterol growth condition. TnDivA identified 15 genes at a cutoff of p ≤ 0.05 and 5 genes at p ≤ 0.01 in common across all three statistical methods (Supplementary_Data_S17). Genes involved in lipid transport and metabolism were common among the different analysis. Several genes were associated with the Mce-family of proteins, believed to be involved in lipid catabolism [82], and other genes were also related to lipid biosynthesis were found to be important for growth on cholesterol (Supplementary_Data_S17).

In the original publication [77], insertion sites within the 5–80% region of protein-coding genes were analyzed followed by read count normalization to equalize mean sequence reads per insertion site. Relative mutant representation was determined using cholesterol-to-glycerol fold changes, and statistical significance was assessed through t-tests for each insertion site across replicates. Genes surpassing a hyperbolic threshold (y = 3.8/x + 0.7) were categorized as important for cholesterol growth. A total of 96 genes were predicted to be important for cholesterol growth. When those predicted genes were compared to the statistically significant results generated by TnDivA, 19 statistically significant genes were identified through log_2_-fold change, 10 genes were identified through Cook’s distance, and 28 and 6 genes were identified through Welch’s t-test at p ≤ 0.05 and p ≤ 0.01 respectively (Supplementary_Data_S18). Overall, 7 genes common in all three analytical methods also overlapped. Among the overlapped genes, the most abundant were those in the Mce-family 1 including those encoding Mce4A, Mce4C, Mce4D, and Mce4F, which are believed to be involved in lipid catabolism, along with many other genes associated with lipid transport and catabolism categories, such as fatty acid biosynthesis enzymes Rv3544c, Rv3546, and Rv3561. Interestingly this same dataset was analyzed by TRANSIT, which identified 28 differentially essential genes (adjusted p-value < 0.05) [75]. Out of those, 8 were essential for glycerol while 20 were essential for cholesterol. Of the genes essential for cholesterol, several genes were reported to belong to the Mce-family of proteins. Thus the original analysis, TRANSIT analysis, and TnDivA analysis all broadly agree on Mce-family proteins and other lipid metabolism proteins as important for growth on cholesterol, but each analysis also has sets of genes unique to each.

## Discussion

To assess the fitness of genes during spaceflight, this study conducted comparative TnSeq of libraries cultured under microgravity (ISS) and on the Ground (normal gravity). Previous comparative TnSeq studies focused on identifying conditionally essential genes but had limitations in the analysis methods. Analyses often considered either unique insertion data [57, 78, 83] or read count (frequency of insertions) data [75, 84]. However, both insertion and read counts have important contribution in identifying the important genes for each condition. Unique insertion sites reflect the ability of a gene to be disrupted, but read counts can reflect the degree to which the mutant can replicate and may be indicative of more subtle fitness changes. Existing tools like ARTIST [79] and Tn-Seq Explorer [78] require high-density transposon libraries, which can be a limitation. The TRANSIT tool [75] that uses a permutation test to compare transposon read counts may exhibit low statistical power with a small number of replicates, potentially providing to misleading results, particularly in the presence of unequal precision in the datasets [85]. TRANSIT is predominantly tailored for mariner-based libraries, but its analytical methods can be extended to Tn5 data, provided that the dataset features sufficient saturation, and assuming insertions’ locations and magnitudes can be treated as effectively random [75]. Notably, Tn5Gaps, an integral component of the TRANSIT suite, is specifically designed to assess gaps or consecutive empty sites between transposon insertions, serving as an indicator of gene essentiality. However, in datasets characterized by high saturation/high noise, the application of Tn5Gaps for resampling analysis to identify conditionally essential genes may yield biased outcomes, potentially misclassifying genuinely essential genes as non-essential. The TSAS tool is designed for analyzing Tn5 datasets and leveraging replicates [76], but its primary mechanism of comparison is through averaged read counts which may not effectively address bias introduced by anomalously high read count inserts within a gene potential biased by anomalously high read count inserts. TSAS also necessitates high-efficiency supercomputers, considerable time for processing and analyzing TnSeq datasets which makes it less suitable for all end-users, particularly when dealing with datasets featuring over a million read counts. Thus, due to all these limitations, previous tools were not the best fit to analyze the data in this study and necessitated a new approach.

This study analyzed differences in the effective density of transposon insertions using a transformation of the Shannon diversity measure. This metric is simple and efficient to calculate, and can be robustly applied to any comparative TnSeq study. Moreover, it is particularly useful for TnSeq study designs with two or more biological replicates [72], which this study leveraged. It should be noted that conducting genome studies in space often requires limited sample size due to logistical and technical constraints which limited the number of replicates, and the number of replicates in this study was further reduced due to contamination. It is not clear where the contaminating reads came from. The fact that 99.9% of reads mapped to the reference genome for GH2_5 and GH2_6 indicated that contamination did not originate from the initial inoculum, as all replicates started with the same library pool and equal number of cells. Also, to ensure accurate results, clean measures were taken at every step of the FPA assembly and genomic DNA extraction process for both ISS and Ground controls. Replicates from each condition were prepared at different times, and once samples were back from spaceflight, genomic DNA from each replicate per condition was individually extracted on different days. All samples were kept separate to avoid any cross-contamination even between replicates. Analysis of reads did not reveal a systemic error, such as an off-by-one pipetting error. Therefore, the origin of the contaminating reads, specifically those mapping to *Agrobacterium tumefaciens*, could not be definitively determined. Unfortunately, given the unusual circumstances of the experimental performance site, the experiment could not be repeated. Therefore, conclusions drawn from these analyses should be considered “presumptive” until individually assessed, though this could be said of any global analysis. The results obtained here should be considered indicative, though not conclusively representative of what could emerge with a larger number of replicates. Subsequently, there remains a need for systematic data verification and reproduction to faithfully confirm the results obtained in this analysis.

The transformed Shannon measure accounts for variability in read distribution between samples, such as cases where some reads are dominated by only a few transposon inserts while in others read distribution is more evenly distributed. This transformation helps to mitigate biases and inconsistencies that may arise from variations in read distribution, ensuring that comparisons are more accurate and reliable. In this study, transformed Shannon diversity indices measured the effective number of transposon sites [69] considering the variability in read distribution of inserts and generating the true and comparable value of transposon diversity per gene. This further accounted for distribution of transposon insertion within a gene by calculating effective density (effective number of transposon sites/gene length), which is used as a normalized measure to compare the effective transposon density per gene between conditions. A higher effective density indicates low fitness contribution for that gene in the given condition, while a lower effective density suggests high fitness contribution of the gene for that condition. Upon analysis of the data, the ISS samples had lower overall effective densities. It is not clear why this was the case. It is unlikely that this phenomenon was solely due to fluctuation in genomic DNA extraction or library preparation processes as it was seen in all replicates. Both conditions underwent the same experimental setup, with the only distinguishable factor being the presence of gravity on the Ground samples and microgravity on ISS samples. Importantly, there was no prior growth before the actual activation of experiment in any of the conditions. All the samples and replicates were assembled in FPA on the ground before being sent to the ISS. No mixing of cells and growth media was performed before the actual time of activation. In space, mixing, incubation, and termination of cultures were performed by astronauts and this was done simultaneously on ground by the researcher. Thus, this procedural consistency should not account for any differences observed, suggesting this is a result of the specific effects of microgravity. One of possible explanation is that ISS samples may simply have not grown as much as Ground samples. Reduced growth of ISS samples could lead to reduced detection of unique transposon sites and/or reduced read counts of detected transposons, leading to lower effective densities. The use of RNAprotect and freezing prevented a post-culturing assessment of growth. It was noted that genomic DNA recovery from ISS samples was generally lower than Ground samples (Table S1 and S2), suggesting that growth aboard the ISS may have been lower. However, visual inspection of samples after culturing revealed detectable growth, and genomic DNA was recovered at sufficient levels for sequencing, suggesting that if growth was impacted, the impact was not large. Without further testing, it remains challenging to pinpoint the exact reason for the global effective density differences between the conditions.

In this study, different methods, including log_2_-fold change, Cook’s distance and Welch’s t-test, were used to identify potentially significant genes. The identified genes have lower effective densities in microgravity and thus are considered to be important for growth in microgravity. Although these methods use different thresholds to analyze the effective transposon density data between conditions, it is suggested that all three methods partially, if not completely, agree with each other in identifying common functions that are important under microgravity. However, the number of potential genes identified varied among the three methods. The number of significant genes identified through Cook’s distance was approximately 10 times more than those identified through log_2_-fold change, and the number of genes identified as statistically significant through Welch’s t- test was approximately 50 times more at p ≤ 0.05 and approximately 30 times more at a cutoff of p ≤ 0.01 compared to log_2_-fold change. The different numbers of predicted genes likely reflect the stringency of the different methods, with log_2_-fold change providing the most stringent predictions and Welch’s t-test at p ≤ 0.05 the most permissive. This suggests that the threshold for detecting significant genes can be adjusted depending on the specific data generated. It is important to note that the log_2_-fold change method can identify potential outliers, this measure does not include a mechanism of statistical significance. Adjusting the significance threshold may be necessary when data sets have different global effective densities, such as observed here. The visual representation of effective densities across the genome clearly showed a distinct difference between the Ground and ISS replicates (Fig. 3), though, the differences were mostly not statistically significant. The global trend of lower effective density in ISS samples essentially added noise to the analyses to find significant genes, but this noise can be overcome. The substantial reduction in the number of significant genes from 1096 (p ≤ 0.05) to 152 (p ≤ 0.01) for AI-1 and from 652 (p ≤ 0.05) to 199 (p ≤ 0.01) for AI-2 suggests that a more stringent threshold is necessary for determining statistical significance when global effective densities are divergent. Furthermore, the removal of low read count replicates had minimal impact on the number of genes detected for log_2_-fold change and Cook’s distance methods, but it significantly affected the number of genes identified through Welch’s t-test and varied based on different cutoffs. Notably, at the stringent cutoff of p ≤ 0.01, the number of genes detected were higher in AI-2 as compared AI-1. This could be attributed to several factors. One of the key factors is the improved data quality in AI-2. By removing the low-quality replicate from the analysis, this helped minimize the variability across the data. In AI-1, the presence of a sample with abnormally lower effective density values may have led to a noisier overall data set, making it harder to achieve more stringent statistical significance. As a result, the number of genes showing significant differences at the stringent cutoff in AI-1 was reduced. On the other hand, AI-2 exhibited lesser variability and a decreased risk of Type 1 errors. With a more prominent difference between the conditions and reduced chances of false positives, higher number of genes with significant differences in fitness were captured at the p ≤ 0.05 cutoff. This may suggest it is better to exclude low read counts to avoid introducing noise or bias into t-test analyses, as their inclusion can lead to differences when they may not be truly significant.

The numbers may differ, but the functional categories of genes identified through all three methods showed similar patterns. The genes predicted to be important were involved in lipid transport and metabolism, secondary metabolism, transcription, translation and energy production. This consistency in the abundance of functional categories across all three methods suggests that the methods used, despite their different thresholds and inconsistent number of gene detections, point towards function-specific rather than gene-specific resolution. In terms of cellular function, hypothetical genes were detected in high numbers to have importance in microgravity. This highlights the need for further research to elucidate their roles and mechanisms in microgravity adaptation. In fact, growth in microgravity may be a way to unravel the cryptic function of these hypothetical genes. Growth in microgravity presents challenges to microorganisms not experienced during normal laboratory culturing and thus may reveal phenotypes in mutants that would not be apparent when grown under standard laboratory conditions. Another cellular function that appears to be important for *N. aromaticivorans* growth in microgravity is lipid metabolism. This COG category was consistently one of the most populous categories in all the analytical methods, with many genes specifically involved in fatty acid biosynthesis represented in the different analyses. Previous space-based bacterial studies have reported altered lipid profiles or changes in pathways associated with lipid metabolism [43, 86, 87]. These studies highlighted the importance of lipid metabolism in microgravity and the observed adaptations through the adjustment in lipid composition and metabolism aim to balance energy conservation and the maintenance of essential cellular functions. Transcriptomic and proteomic analyses of *E.coli* after 17 days of spaceflight revealed associated with phospholipid biosynthesis, metabolism, organophosphate metabolism, lipid biosynthesis and cellular lipid metabolism [43]. A study on *Staphylococcus aureus* using simulated microgravity identified changes in fatty acid profiles and an increase in susceptibility to membrane-targeting antibiotics [88]. The importance of lipid metabolism in bacterial response to microgravity appears to be a growing trend that the results from this present study support.

While the exact reason for lipid metabolic changes is unclear, the microgravity environment of spaceflight is thought to induce stress in bacteria, and bacteria are known to adjust their membrane lipid composition in response to stressful environmental conditions by modulating the relative amounts of different types of lipids and the degree of unsaturation of fatty acyl chains [89]. For example, acetyl-CoA carboxylase (ACC) is a critical multi subunit enzyme responsible for initiating the essential first step in fatty acid synthesis [90], and acyl-CoA dehydrogenase (ACD) is a critical enzyme in lipid metabolism that introduces unsaturation into fatty acids, with the cofactor acyl-CoA converting to enoyl-CoA [91]. Gene *saro_1903* is an ACC and *saro_3691* and *saro_3748* are ACDs, all of which were found to be important for growth in microgravity in this study. Similarly, maintaining membrane integrity is vital for cell survival, especially in response to environmental stresses which is known to impact lipid profiles and membrane fluidity. Fatty acid desaturases enable the synthesis of unsaturated fatty acids and play a crucial role in enhancing membrane fluidity, which is vital for adapting to changes in membrane properties caused by microgravity [92]. Gene *saro_3890* is a fatty acid desaturase found in this study. Additionally, these unsaturated fatty acids provide resistance against oxidative stress, a potent stressor during spaceflight, by reducing the susceptibility of cell membranes to oxidative damage [93]. Short-chain dehydrogenase/reductase (SDRs) enzymes have diverse molecular functions that include playing an essential role in lipid metabolism [94] and lipid A modification in Gram-negative bacteria [95]. Enoyl-ACP reductases utilize NADH and catalyze the final and rate-determining step in elongation cycle of type II bacterial fatty acid synthesis, acting as a key regulatory protein in this process [96]. The identification of multiple SDRs including *saro_3600*, *saro_1564*, *saro_2767* and *saro_3083* as low-fitness genes under microgravity conditions underscores their substantial importance in addressing the unique challenges posed where altered fluid dynamics and membrane integrity become stressful, and these functions become paramount for survival.

Similarly, the increased representation of genes associated with secondary metabolism suggests that the production of specialized metabolites which are not directly involved in basic cellular functions plays a crucial role in adaptation, defense, or signaling under stressful conditions. The detected enzymes have distinct roles including participating in the catabolism of aromatic hydrocarbons, detoxifying harmful byproducts, and facilitating their transport. 2-chlorobenzoate 1,2-dioxygenase is particularly important for the degradation of dichlorobenzoates (DCBs), an aromatic compound that can be degraded and utilized as an alternative carbon source for bacterial growth [97, 98]. The importance of this gene (*saro_0539*) in *N. aromaticivorans* may suggest that it searches for alternative carbon sources under microgravity. The overexpression of genes necessary for the transport of alternative carbon sources into bacterial cells, even when carbon sources are not available has been suggested by many studies conducted in the ISS [49]. Additionally, enzymes like catechol 2,3-dioxygenase, lactoylglutathione lyase family proteins, or glyoxalase/bleomycin resistance protein/bleomycin resistance proteins play a crucial role in detoxifying cytotoxic and mutagenic compounds like methylglyoxal [99] and bleomycin [100]. The production of methylglyoxal is often linked to an imbalance between the rate of carbon acquisition and the capacity of the lower segment of glycolysis [101]. It is also reported to be originated from the degradation of acetone and threonine [99]. Our finding of these enzymes may suggest that *N. aromaticavorans* experienced central carbon metabolism stress in microgravity that necessitated these detoxification enzymes. Interestingly, there is a strong connection between lipid biosynthesis and secondary metabolite biosynthesis as these two processes often draw on the same precursor pool [49]. Furthermore, these precursor pools are tied to central metabolic pathways (e.g. acetyl-CoA), and two other common COG categories that arose in the analyses were carbohydrate metabolism and coenzyme metabolism. Taken together, these results may suggest that the increased stress of growth in microgravity leads to lipid remodeling and altered secondary metabolite production, which then puts strain on central metabolic processes such that mutations in them lead to reduced fitness.

The increased representation of transcription genes is not surprising. It suggests the presence of specific regulators coordinating the pathways of adaptive response to stress by important genes for microgravity. Notably, Lrp/AsnC families of transcriptional regulators encompasses proteins involved in amino acid metabolism, regulation of transport processes, or cell morphogenesis [102]. AsnC is a specific gene regulator whose activity is triggered by asparagine binding. The increased uptake of asparagine and involvement of asparagine pathway for the effective countermeasure against a stress linked to modified nutrient utilization capabilities has been reported under simulated microgravity condition [103]. Transcription regulators like TetR are responsible for regulating efflux pump expression and various cellular processes [104], highlighting their importance in the stressful microgravity condition where excretion of toxins would be an important response for cell survival. LysR-type transcriptional regulators (LTTRs), which govern a diverse set of genes related to metabolism and quorum sensing [105], further play a pivotal role in adaptive responses to microgravity-induced stress [9]. Likewise, importance of AraC family members, which predominantly are involved in the regulation of glycerolipid metabolism, and, osmotic stress response, and MarR family members often antibiotic resistance regulators, can be explained by microbial stress responses. The detected genes strongly suggest that disruption of these core physiological processes impact the survival strategies and different pathways involved for the survival of *N. aromaticivorans* under stressful conditions.

Perhaps as interesting as the genes significant in these analyses are the genes that were not significant. Previous studies of bacterial responses to spaceflight identified stress regulons involved in DNA damage, oxidative stress, and cell envelope response (often associated with radiation exposure) being upregulated after exposure to microgravity [8, 106]. This study identified few genes associated with these processes. Similarly, a strong correlation between motility and the effect of space flight on the cell growth have been reported [26, 107], but low number of genes at p ≤ 0.05 and no genes at p ≤ 0.01 related to cell motility in this study indicates that this physiological response may be less critical for *N. aromaticivorans* survival or adaptation to the microgravity environment. One of the most important bacterial responses to microgravity is increased biofilm formation, which can have catastrophic effect on flight infrastructure. This study found no genes associated with biofilm formation. So why were these canonically important processes not detected as important in this study? One possibility is that it simply is a consequence of the experimental design. The reduced representation of genes involved in DNA repair may indicate that the level of cellular damage due to radiation might not be as significant as initially anticipated within the protective enclosed environment of an incubator aboard the International Space Station. Culturing in a relatively rich growth medium may not require cellular motility to scavenge nutrients even in a diffusion-limited environment, or the open space of the FPA was not conducive to establishing a biofilm. Alternatively, it is possible that *N. aromaticivorans* has a different response to microgravity than the model organisms tested before. As stated, bacterial responses to microgravity are varied and often inconsistent. These results highlight the need to further experiment with non-model organisms under different experimental conditions.

While TnSeq has been used as a methodology for some time, there is no universally accepted method for TnSeq data analysis, and there is even less consistency in regards to comparative TnSeq. What works under one scenario may not work under another. In this study, TRANSIT proved an ineffective tool for data analysis, unable to identify any genes as essential under any condition. The most likely explanation for this result is that tools like Tn5Gaps that identify essentiality based on gaps between transposon insertions are limited on datasets that are highly saturated or have low frequency insertions in otherwise essential genes (i.e. noise). TSAS analysis of the data produced conditionally essential genes in broad agreement with TnDivA results, but TSAS is more susceptible to biases from aberrant high read count inserts and is computationally expensive. There was also broad agreement on genes important for growth on cholesterol by *M. tuberculosis* between the original comparison method, TRANSIT analysis, and TnDivA analysis. Yet, every analysis identified genes not found in the other analyses. The true conditionally essential genes are likely to be found in the overlap of the analyses. This highlights the importance of performing and comparing multiple analytical methods.

Here we offer TnDivA as a robust comparative TnSeq tool. TnDivA is a more mathematically accurate representation of transposon diversity, is resilient to noise in the data, is computationally cheap, is amenable to multiple downstream analytical methods, and most downstream methods were resistant to the inclusion of a low-quality replicate. TnDivA offers the advantage of accommodating different library saturation and provides the freedom to customize cutoff criteria based on the characteristics of the data, enabling more precise and context-specific analysis.

In conclusion, the analysis of gene fitness in *N. aromaticivorans* reveals a broad impact of microgravity on larger biological processes identified through different comparative analysis tools. The patterns observed in gene fitness and COG annotations indicate a higher abundance of genes associated with metabolism, transcription, translation, and energy production, which are critical for survival and adaptive strategies in response to microgravity. The observed impacts on metabolism genes, both primary and secondary, highlight the global physiological effects of stress in microgravity. It is important to note that these interpretations are specific to *N. aromaticivorans* and may vary for other bacterial species. These findings contribute to our understanding of the unique adaptations required for microgravity conditions and can guide the development of strategies to enhance or diminish organism resilience in space missions.

### Electronic supplementary material

Below is the link to the electronic supplementary material.

Supplementary Material 1

Supplementary Material 2

Supplementary Material 3

Supplementary Material 4

Supplementary Material 5

Supplementary Material 6

Supplementary Material 7

Supplementary Material 8

Supplementary Material 9

Supplementary Material 10

Supplementary Material 11

Supplementary Material 12

Supplementary Material 13

Supplementary Material 14

Supplementary Material 15

Supplementary Material 16

Supplementary Material 17

Supplementary Material 18

Supplementary Material 19

## Data Availability

The TnSeq dataset generated during this study is available in the NCBI|NLM|NIH under BioProject accession number PRJNA998419. The link below includes all the TnSeq data for both the experimental and control samples used in this study, which is now publicly available. https://www.ncbi.nlm.nih.gov/bioproject/PRJNA998419. TnDivA is an end user-friendly analysis for comparing TnSeq datasets, that can be used for any kind of transposon library. Written as R code, it’s compatible with Linux, Mac, and Windows, but requires specific R packages. The source code and documentation can be found on its GitHub repository: TnDivA (https://github.com/gayatri-101/TnDivA/tree/main).
