# Supplementary material for: Whole genome-scale assessment of gene fitness of Novosphingobium aromaticavorans during spaceflight
Source: BMC Genomics. 2023 Dec 16;24:782. doi: 10.1186/s12864-023-09799-z (PMC10725011; doi:10.1186/s12864-023-09799-z)
Supplement: Supplementary file 2 — Supplementary Material 2 [file 12864_2023_9799_MOESM2_ESM.docx]

| **PCR1** |  |  |  |  |  |  |
| --- | --- | --- | --- | --- | --- | --- |
| **Forward Primers** |  |  |  |  |  |  |
| **Primer** | **PCR** | **Seq** |  |  | **Tm** | bp from end |
| EZTn PCR1 For | PCR1 | aggatcagatcacgcatcttc |  |  | 61.8 | 150 |
| EZTn PCR1B For | PCR1 | cctgcaggcatgcaag |  |  | 60.7 | 60 |
|  |  |  |  |  |  |  |
|  |  |  |  |  |  |  |
| **Reverse Primers** |  |  |  |  |  |  |
| **Primer** | **PCR** | **Seq** |  |  | **Tm** | bp from end |
| EXTn PCR1 Rev | PCR1 | aggttgaactgctgatcttc |  |  | 60 | 165 |
| EZTn PCR1B Rev | PCR1 | gaactgctgatcttcggatc |  |  | 60.2 | 160 |

| **PCR2** |  |  |  |  |  |  |
| --- | --- | --- | --- | --- | --- | --- |
| **Primer** | **PCR** | **Seq** |  |  | **Tm** | bp from end |
| EZTn PCR2 For-Ill | PCR2 | GTTCAGACGTGTGCTCTTCCGATCTcacatctcaaccctgaagc |  |  | 61 | 30 |
| EXTn PCR2 Rev-Ill | PCR2 | GTTCAGACGTGTGCTCTTCCGATCTcgatgatggttgagatgtg |  |  | 58.1 | 32 |
