# Supplementary material for: Whole genome-scale assessment of gene fitness of Novosphingobium aromaticavorans during spaceflight
Source: BMC Genomics. 2023 Dec 16;24:782. doi: 10.1186/s12864-023-09799-z (PMC10725011; doi:10.1186/s12864-023-09799-z)
Supplement: Supplementary file 12 — Supplementary Material 12 [file 12864_2023_9799_MOESM12_ESM.docx]

**This file provides the information of most abundant COG categories of the important genes identified through AI-1 data analysis**

**Outlier genes through log_2_-fold change for AI-1 data set**

Among the 22 genes identified as outlier (>1 log_2_-fold) in AI-1, the most abundant COG category was hypothetical genes, comprising 7 genes. Out of the remaining genes, most represented functional categories were lipid metabolism and transport with 3 genes and carbohydrate transport and metabolism with 2 genes. Additionally, there was 1 gene involved in secondary metabolite biosynthesis, transport, and catabolism, 1 gene involved in energy production, and 1 gene in the translation, ribosomal structure and biogenesis category.

**Influential gene through Cook’s distance for AI-1 data set**

For AI-1, out of 200 genes identified as significant outlier genes, 72 were hypothetical genes, 4 genes had general function prediction and 3 genes had unknown function. The most represented functional categories were lipid transport and metabolism with 8 genes, secondary metabolite biosynthesis with 7 genes, transcription with 10 genes, translation, ribosomal structure, and biogenesis with 7 genes, carbohydrate transport and metabolism with 7 genes and replication, repair and recombination with 9 genes

**Statistically significant genes identified through welch’s t-test**

**p-value ≤ 0.05**

In AI-1, out of 1096 genes identified as significant at p ≤ 0.05, 235 genes were hypothetical genes, 42 were genes with unknown function, and 108 genes were annotated as general function prediction (Figure 8A). In terms of defined function, the most represented predicted groups included 94 genes involved in lipid transport and metabolism, 80 genes involved in secondary metabolite biosynthesis, transport, and catabolism, 79 transcription-associated genes, and 72 genes involved in energy production and conservation

**p-value ≤ 0.01**

For AI-1, out of 152 significant genes identified, 46 were hypothetical, 9 as unknown function, and 14 were assigned as general function prediction. Out of genes with predicted functions, the most represented groups were 12 genes associated with lipid transport and metabolism, 11 genes related to secondary metabolite biosynthesis, transport, and catabolism, and various groups with 10 genes, such as carbohydrate metabolism and translation.

**Statistically significant influential genes identified through log_2_-fold change, Cook’s distance and Welch’s t test at both p-value ≤ 0.05 and p-value ≤ 0.05** **for AI-1 dataset**Top of Form

Out of 200 influential genes detected in AI-1 as determined by Cook’s distance, only 37 genes were detected to be statistically different between conditions at p ≤ 0.05 cutoff using Welch’s t-test, and included just 1 gene identified as an outlier from log_2_- transformation. The more stringent p ≤ 0.01 cutoff identified a total of 12 genes that had significant differences in their effective densities, with no genes identified as an outlier from log_2_-fold change

**COG categories of common genes identified through all three statistical methods for AI-1 dataset**

Among the total 37 outlier genes from Cook’s distance found in the AI-1 dataset at p ≤ 0.05, 12 were classified as hypothetical genes, 3 were involved in lipid metabolism and transport, 6 were associated with secondary metabolite biosynthesis, transport, and catabolism, 4 were transcription-related, 2 were translation-related, and 1 gene each was related to carbohydrate transport and metabolism. One significant gene was also found common for log_2_-fold change, Cook’s distance and Welch’s t-test at p ≤ 0.05 associated with lipid metabolism and transport

Similar results were found when comparing genes significant in the Cook’s distance method to Welch’s t-test results significant genes from AI-1 at the p ≤ 0.01 cutoff
