# Supplementary material for: Whole genome-scale assessment of gene fitness of Novosphingobium aromaticavorans during spaceflight
Source: BMC Genomics. 2023 Dec 16;24:782. doi: 10.1186/s12864-023-09799-z (PMC10725011; doi:10.1186/s12864-023-09799-z)
Supplement: Supplementary file 19 — Supplementary Material 19 [file 12864_2023_9799_MOESM19_ESM.docx]

| **Ground Samples** | Buffer Type | DNA Concentration (ng/µl) | 260/280 | 260/230 |
| --- | --- | --- | --- | --- |
| GH-2.5 | Nuclease free water | 297.0ng/µl | 2 | 1.78 |
| GH-2.6 | Nuclease free water | 364.5ng/µl | 2 | 1.5 |
| GH-2.7 | Nuclease free water | 87.8ng/µl | 2 | 1.4 |
| GH-2.8 | Nuclease free water | 81.9ng/µl | 2 | 1.5 |

**Table S1**: Genomic DNA concentration of Ground replicates

**Table S2**: Genomic DNA concentration of ISS replicates

| **ISS Samples** | Buffer Type | DNA Concentration (ng/µl) | 260/280 | 260/230 |
| --- | --- | --- | --- | --- |
| ISS-2.5 | Nuclease free water | 168.5ng/µl | 2 | 1.78 |
| ISS-2.6 | Nuclease free water | 35.6.ng/µl | 1.8 | 1.74 |
| ISS-2.7 | Nuclease free water | 166.6ng/µl | 1.9 | 1.66 |
| ISS-2.8 | Nuclease free water | 138.0ng/µl | 1.8 | 1.45 |
